# The association between the MIND diet and cognitive health in middle-aged and older adults: A systematic review

**DOI:** 10.1016/j.jnha.2025.100630

**Published:** 2025-07-10

**Authors:** Jenny Hiu Wai Tse, Queenie Pui Sze Law, Jenny Tsun Yee Tsang, Lorna Kwai Ping Suen, Stefanos Tyrovolas, Rick Yiu Cho Kwan

**Affiliations:** a School of Nursing, Tung Wah College, Hong Kong SAR, China; b School of Nursing and Health Sciences, Hong Kong Metropolitan University, Hong Kong SAR, China; c Department of Nutrition and Food Studies, George Mason University, Virginia, USA; d Research, Innovation and Teaching Unit, Parc Sanitari Sant Joan de Déu, 08830 Sant Boi de Llobregat, Spain; e Instituto de Salud Carlos III, Centro de Investigación Biomédica en Red de Salud Mental, Centro de Investigación Biomédica en Red de Salud Mental (CIBERSAM), 28029 Madrid, Spain

**Keywords:** MIND diet, Mediterranean, DASH, Cognitive health, Alzheimer's disease, Dementia, Middle-aged adults, Older adults, Dietary interventions, Memory, Executive function

## Abstract

**Background:**

Cognitive decline, a natural aspect of aging, is linked to negative outcomes like increased mortality and social isolation. The Mediterranean-DASH Intervention for Neurodegenerative Delay (MIND) diet, blending the Mediterranean diet and the Dietary Approaches to Stop Hypertension (DASH) diet elements, aims to slow cognitive decline and reduce dementia risk. Secondary analyses of population studies and randomized controlled trials (RCTs) show mixed results on the MIND diet's effectiveness in improving cognitive health. Existing reviews have explored the MIND diet's impact on cognitive health, though their focus can be broad or narrow. Our review offers an updated perspective by specifically targeting dementia risk and clinical cognitive function, integrating new studies for enhanced insights into clinical practice and research.

**Methods:**

This review followed the Preferred Reporting Items for Systematic Reviews and Meta-Analyses (PRISMA) 2020 and the Synthesis Without Meta-analysis (SWiM) guidelines and was registered in PROSPERO (CRD42023391972). We included quantitative studies on middle-aged and older adults (mean age >40 years) examining MIND diet adherence and cognitive health, excluding non-original research. A systematic search was conducted in five databases from March 2023 to March 2024 using relevant search terms. Data were extracted and assessed for bias by multiple reviewers using Joanna Briggs Institute (JBI) tools. Heterogeneous data were synthesized using SWiM guidelines, focusing on cognitive function outcomes, with results presented in tables and figures.

**Results:**

The search over five databases identified 898 articles, with 26 meeting the inclusion criteria. A hand search added 13 more, totaling 39 articles from 14 countries, including cohorts, cross-sectional, RCTs, and case-control studies. Most studies were conducted in the United States of America (USA), published between 2015 and 2024. Participant numbers ranged from 37 to 120,661, with follow-ups from 3 months to 24 years. Some studies explored more than one correlation. Of the studies, 14 out of 19 articles explored MIND diet adherence and global cognitive function, showing positive results. 10 out of 11 studies investigated MIND diet adherence and dementia/Alzheimer’s risk, showing positive associations. 16 out of 18 articles examined the MIND diet's effect on domain-specific cognitive functions, with favorable outcomes.

**Discussion:**

This systematic review reveals the significant promise of the MIND diet in enhancing cognitive health, specifically in global cognition, memory, and executive function. While observational studies strongly advocate for its inclusion in clinical guidelines to prevent and manage Alzheimer's disease (AD) and dementia, results from RCTs are mixed, suggesting further investigation is needed. The use of PRISMA and SWiM guidelines ensures robust and transparent findings, categorizing cognitive outcomes into critical areas for a holistic insight. Despite the effectiveness of alternative methods, such as MIND diet questionnaires, for adherence assessment apart from FFQ, variability in study populations, interventions, and scoring methods complicates pinpointing an optimal MIND score. This underscores the importance of standardized methodologies to refine dietary recommendations and consolidate the diet's cognitive health benefits across various populations.

## 1 Introduction

Cognitive function declines with age due to various neural mechanisms. [1] Slower cognitive decline is associated with negative health outcomes in older people, including lower mortality, social isolation, and sensory impairment [[2], [3], [4]]. Over 55 million people worldwide have dementia, characterized by declines in memory and thinking, with Alzheimer's disease accounting for 60–70 % of cases. Nearly 10 million new cases arise annually, making dementia the seventh leading cause of death globally and a major contributor to disability among older adults. [5] Brain ageing varies among individuals based on a spectrum of risk factors, including genetics, lifestyle, and diseases [6]. Midlife brain age is associated with accelerated biological ageing and cognitive decline [7]. Reducing modifiable risk factors through a life-course approach to promote cognitive health is supported by strong evidence and advocated by WHO [8,9]. Dietary patterns remain key factors affecting mid- and late-life cognitive health, prompting research into recommendations for older people [8,10].

MIND diet blends the Mediterranean and DASH diets, modified according to diet-dementia findings. [11] Mediterranean diet is associated with various health outcomes in older people. When combined with the DASH diet, the MIND diet demonstrates a stronger association with cognitive health than either diet alone [[12], [13], [14], [15]]. The MIND diet classifies food into 15 types, divided into encourage-intake (i.e., olive oil, fish, whole grains, berries, green leafy vegetables, other vegetables, nuts, beans, poultry, and wine) and limited-intake (i.e., butter and margarine, cheese, red meat and meat products, fast and fried foods, and pastries and sweets) groups. [16] Proposed by Morris et al. in 2015, the MIND diet potentially slows cognitive decline and reduces Alzheimer’s disease incidence [11,17]. Better adherence is linked to these outcomes [11,17,18].

In the past decade, after Morris group’s publication, [11,17] many secondary data analyses (e.g., Nurses’ Health Study, Framingham Heart Study, Rotterdam Study, Whitehall II Study, Health and Retirement Study) explored MIND diet’s effect on cognitive health [[19], [20], [21], [22], [23], [24]]. Most findings support that adherence correlated with better cognitive function, such as verbal memory [19], global cognitive function, and larger total brain volume [20], decreased dementia risk [21], better cognitive function [22], and reduced risk of cognitive impairment [23]. RCTs were conducted in different populations, including healthy obese women [25], and overweight older people without cognitive impairment but with a family history of dementia. [26] However, they yielded conflicting results regarding the MIND diet's efficacy^25^, [26].

Several systematic reviews attempted to conclude the effect of the MIND diet on cognitive health. [18,27,28] While they explored the diet impacts, existing articles are often too broad, addressing overall brain aging, or too specific, concentrating solely on cognitive decline. Other reviews did not systematically identify literature or target the MIND diet, leading to selection bias [12,29]. Our review specifically focuses on dementia risk and clinical cognitive function, providing a nuanced perspective on the clinical and research implications. It also integrates new studies not included in previous reviews, offering updated evidence that enhances understanding of the MIND diet’s role in cognitive health.

## 2 Methods

This study was performed according to the PRISMA guideline 2020 [30] and the SWiM extension [31]. The study protocol was registered in the PROSPERO, International Prospective Register of Systematic Reviews, identifier: CRD42023391972.

### 2.1 Eligibility criteria

The search strategy aimed to identify research articles reporting on the relationship between MIND diet and cognitive health in middle-aged and older people. Inclusion and exclusion criteria were developed based on PI/ECO and study design to guide the selection of relevant articles.

#### 2.1.1 Inclusion criteria

1 Population: middle-aged or older adults in the study population, as defined as mean age of study population >40 years,
2 Intervention/Exposure: having a group that was exposed or had higher adherence to the MIND diet,
3 Comparison: having a group that was unexposed or had lower adherence to MIND diet,
4 Outcomes: cognitive health, including prevalence of cognitive degenerative diseases and cognitive functions, and
5 Study design: Quantitative studies, including experimental studies (e.g., randomized controlled trials), and observational studies (e.g., cross-sectional studies, case-control studies, cohort studies)

#### 2.1.2 Exclusion criteria

1 Non-original research (e.g., systematic review, letter to editor, literature review, commentary).
2 Papers not primarily evaluating the association between the MIND diet and cognitive health outcomes.
3 Studies lacking inferential statistics or those that only describe the values of the MIND diet and cognitive outcomes as part of a secondary descriptive analysis.

### 2.2 Information sources

To identify relevant peer-reviewed articles, a systematic search was conducted in five electronic databases, including CINAHL Complete, PubMed, Cochrane Library, Web of Science, and EMBASE, between March 2023 and March 2024.

### 2.3 Search strategy

The search language was based on Medical Subject Headings (MeSH) terms. The descriptors used were “MIND diet”, “Neurodegenerative Delay diet”, “middle age”, “middle-age”, “middle aged”, “middle-aged”, “adult*”, “older adult*”, “older person*”, “older people”, “elderly”, “senior”, “dementia”, “Alzheimer's Disease”, “mild cognitive impairment”, “cognitive dysfunction*”, “cognitive impairment*”, and “cognitive disorder”. The Boolean operators AND and OR were employed to refine and expand the search, respectively. The descriptors and search syntax used in each database are provided in Appendix I. This review specifically focuses on articles published in or after 2015 when the MIND diet was initially introduced by Morris et al. (2015). [11] After identifying eligible articles following the study selection section criteria, a manual search was conducted on the reference lists of these articles to identify any additional potentially eligible articles.

### 2.4 Selection process

After executing the search strategy, the identified articles were imported into the reference management software, Rayyan. [32] Duplicates were then removed using the software's built-in “detect duplicate” function. The title and abstract of all identified articles were screened independently by two reviewers by applying the eligibility criteria. Full texts of the potentially eligible articles were retrieved, and the authors were contacted when the full texts were not available. The identified articles were subjected to full-text screening using the same procedure. Discrepancies between reviewers were resolved through team discussion.

### 2.5 Data collection process

The data of each eligible article was extracted by three independent authors (JHWT, JTYT, and QL) and was recorded using a Microsoft Excel (version 2020) data extraction table developed for this review. If there were any disagreements over the extraction of data, the three independent authors invited another author (RK) to discuss the matter according to the pre-defined nature of the data items. In the case of queries, attempts were made to contact the authors of the studies for clarification.

### 2.6 Data items

The data items included general information about the study profiles, such as author, publication year, and country, along with specific information about the study population (e.g., number and age of participants), intervention (e.g., dietary patterns, MIND diet intake & adherence assessment, and follow-up duration), comparison (e.g., condition and control group details), outcomes (e.g., global cognitive function, domain-specific cognitive functions, and prevalence of cognitive degenerative diseases), and study design.

### 2.7 Study risk of bias assessment

The risk of bias and quality assessment of the included studies were conducted independently by the three reviewers using the JBI’s critical appraisal tools. [33] JBI's specific appraisal checklists corresponding to the study designs were employed, such as those for cohort studies, cross-sectional studies, case-control studies [34], and randomized controlled trials [35]. The checklists used for the quality assessment are included in Appendix II for reference. These checklists comprehensively evaluate various aspects, including participant selection, intervention design, measurement of exposure and outcomes, and statistical analytic methods, resulting in a score that reflects the quality of the included studies. Based on the discussion between the reviewers and with reference to a previous study using JBI critical appraisal tools, [36] The cut-off JBI scores for classifying the studies as "good" or "poor" quality are specific to the study designs: 7 out of 11 for cohort studies, 5 out of 8 for cross-sectional studies, 8 out of 13 for RCTs, and 6 out of 10 for case-control studies. All selected studies were included in this review for data synthesis regardless of their JBI scores.

### 2.8 Data synthesis

Given the heterogeneity in the study population, intervention conditions, outcome measurements, and study designs, conducting a meta-analysis was considered unsuitable. Instead, the SWiM guidelines [31] were employed to transparently synthesize the data from the articles included. The SWiM items facilitate the grouping of studies and provide guidance on reporting standardized metrics used for synthesizing findings. Accordingly, we followed these steps:

1 Summarized the characteristics of each study, including population information, intervention implementation, outcome measures, study design, and methodological quality. We determined which studies were similar enough to be grouped together for data synthesis, such as those with similar outcome measures and study designs.
2 Identified the available data to be used as standardized metrics for synthesis. These data encompassed the direction of effect (e.g., positive and negative results) and the significance of the results (e.g., p-value).
3 Synthesized the characteristics of the studies. The direction of effect data was synthesized by the vote-counting method.
4 Prioritized results for synthesis. In this study, the synthesis of data focused on outcome measures that are crucial for addressing the aim of this review, namely the risk of cognitive degenerative diseases, global cognitive function, and domain-specific cognitive function. These measures were given priority as they provide the most significant information for our research.
5 Investigated the heterogeneity of the reported effects. We identified the study design (e.g., RCT, cross-sectional study) and measurement methods (e.g., type of instrument used) among the included studies in each outcome measure analysis.
6 Assessed the certainty of synthesized findings by reporting the number of studies and participants, the consistency of effects across studies, the risk of bias in the included studies, and the direct relevance of the included studies to the corresponding research aim.
7 Presented the above data in tables and figures as appropriate.

## 3 Results

### 3.1 Study selection

**Fig. 1:**
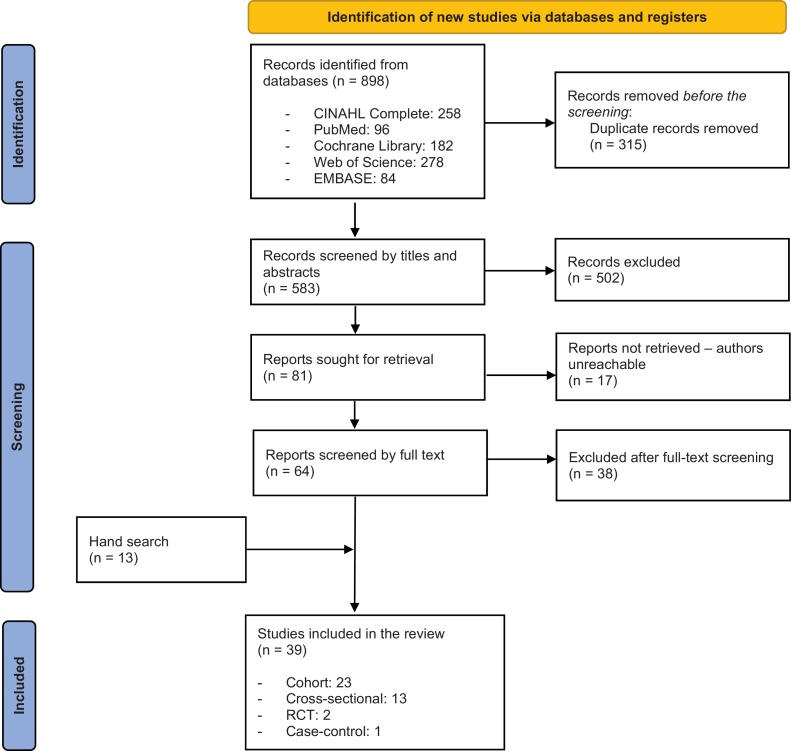
PRISMA flowchart of the identified and screened studies.

Two reviewers independently screened the articles based on the pre-established eligibility criteria. Any discrepancies between the reviewers were resolved through discussion. The selection process of the included studies is detailed in Fig. 1, represented by the PRISMA flow diagram. The initial search yielded 898 articles. After the removal of duplicates, 583 articles remained, which were then screened by their titles and abstracts for relevance. From this screening, 502 articles were not eligible and excluded, resulting in 81 articles being considered for full-text assessment. Of these, 64 full-text articles were either eligible or of uncertain eligibility and were retrieved. Unfortunately, despite efforts to contact the authors, 17 full-text articles could not be obtained. After a thorough screening, 38 articles did not meet the inclusion criteria and were subsequently excluded. Additionally, a hand search identified 13 additional articles. Ultimately, a total of 39 articles were identified and subjected to quality assessment.

### 3.2 Study characteristics

A total of 39 studies from 14 countries were reviewed. Key characteristics of the included studies, categorized by study designs and then sequenced by year of publication, are detailed in Table 1. The majority of the included studies employed a cohort study design (n = 23/39, 59.0%), followed by cross-sectional studies (n = 13/39, 33.3%). The remaining studies comprised randomized controlled trials (n = 2/39, 5.1%) and case-control studies (n = 1/39, 2.6%). The studies eligible for this analysis were published between 2015 and 2024. Geographically, although most studies were conducted in the USA (n = 17/39, 43.6%), the remaining studies included participants from various countries around the world, including the China (n = 3/39, 7.7%), United Kingdom (UK) (n = 3/39, 7.7%), Spain (n = 2/39, 5.1%), Brazil (n = 2/39, 5.1%), France (n = 2/39, 5.1%), Australia (n = 2/39, 5.1%), Germany (n = 1/29, 3.4%), Iran (n = 2/29, 3.4%), Italy (n = 1/29, 3.4%), the Netherlands (n = 1/39, 2.6%), Greece (n = 1/39, 2.6%), Sweden (n = 1/39, 2.6%) and Israel (n = 1/39, 2.6%). The number of participants across the studies varied significantly, ranging from 37 to 120,661, with mean ages spanning from 48 to 90.8 years. Follow-up durations also varied, with cohort studies ranging from 2 to 24 years and randomized controlled trials ranging from 3 months to 3 years.

**Table 1:** Characteristics of included studies.

No.	First author (year), country	Study type	Population size	Age Mean ± SD	Health Status at Baseline	Follow-up duration	Intervention / Exposure (measures of intake and adherence)	*Comparison	#Outcome (Assessment methods)
	**Cohort studies**								
1	Morris (2015), [17] USA	Cohort Study	923	Mean not specified, age range: 58−98	Without AD	Average 4.5 years Cognitive assessment: -Annual evaluation -At least twice	15-MIND diet (144-item FFQ, assessed intake over the previous 12 months; MIND diet score 0−15 catergorized into tertiles)	Different MIND diet adherence groups: - Tertile 1: 2.5−6.5, mean = 5.6 - Tertile 2: 7.0−8.0, mean = 7.5 - Tertile 3: 8.5−12.5, mean = 9.6 Range: 2.5–12.5 Average: 7.4	1. Risk of dementia or Alzheimer’s disease (by experienced clinician; structured neurological exam; medical history; cognitive tests; algorithmic rating)
2	Morris (2015), [11] USA	Cohort Study	960	81.4 ± 7.2	Without dementia	mean = 4.7 years MIND diet: Complete FFQ annually. Cognitive assessment: Annual clinical cognitive testing.	15-MIND diet (144-item FFQ; assessed intake over the previous 12 months; MIND diet score 0−15 catergorized into tertiles)	Different MIND diet adherence groups (continuous): - Tertile 1: 2.5−6.5, mean = 5.6 - Tertile 2: 7.0−8.0, mean = 7.5 - Tertile 3: 8.5−12.5, mean = 9.6 Range: 2.5–12.5 Average: 7.4	1. Global cognitive function 2. Domain-specific cognitive functions (episodic memory, working memory, semantic memory, visuospatial ability, and perceptual speed)
3	Berendsen (2018), [19] USA	Cohort Study	16,058 (Female only)	74.3 ± 2.3	Without stroke	24 years •FFQ x 5 •Cognitive health assessmenet x 4	15-MIND diet (116-item FFQ; MIND diet score 0−15 categorized into quintiles)	Different MIND diet adherence groups: - Quintile 1: 2.6−5.4 - Quintile 2: 5.5−6.1 - Quintile 3: 6.1−6.7 - Quintile 4: 6.7−7.3 - Quintile 5: 7.4−11.0	1. Global cognitive function 2. Domain-specific cognitive functions (TICS; Verbal (Episodic) Memory; Language and Executive Function; Working Memory and Attention)
4	Shakersain (2018), [37] Sweden	Cohort Study	2223	Men: 69.5 ± 8.6 Women: 71.3 ± 9.1	Without dementia	6 years MIND diet and cognitive assessment: Baseline: March 2001 to June 2004; Follow-up evaluation every 3 years (age <78) or 6 years (aged ≥ 78)	14-MIND diet (The intake of nuts and olive oil was replaced by vegetable oil) (98-item FFQ; assessed intakes over the past 12 months; MIND diet score 0–66 classified into three categories.)	Different MIND diet adherence groups: - Tertile 1 (lowest) - Tertile 2 (moderate) - Tertile 3 (highest)	1. Global cognitive function (MMSE)
5	Adjibade (2019), [38] France	Cohort Study	6,011	64.4 ± 4.3	Without SMC, depression or treatment with antidepressants	Mean = 6 years MIND diet: Baseline and then twice a year. Cognitive assessment: From 2 years of follow-up, questionnaire was sent every 2 years: T1: 2011 T2: 2013 T3: 2015 T4: 2017	15-MIND diet (Web-based 24-h dietary record tool, completed three over a 2-week period; MIND diet score 0−15 categorized into tertiles)	Different MIND diet adherence groups: - Tertile 1: 1.0−5.0 - Tertile 2: 5.5−6.5 - Tertile 3: 7.0−11.5	1. Risk of dementia or Alzheimer’s disease (SMC - possible precursor of MCI and AD) (37-item self-administered CDS questionnaire to assess daily deficiencies; lapses of attention or memory; related functions)
6	Cherian (2019), [39] USA	Cohort Study	106	82.8 ± 7.1	Had clinical history of stroke	Average 5.9 years Completed at least two annual cognitive assessments	15-MIND diet (144-item FFQ, assess intake over the prior 12 months; MIND diet score 0−15 categorized into tertiles)	Different MIND diet adherence groups: - Tertile 1: median 6.0 - Tertile 2: median 7.5 - Tertile 3: median 9.5	1. Global cognitive function 2. Domain-specific cognitive functions (episodic memory; semantic memory; working memory; perceptual orientation; perceptual speed)
7	Hosking (2019), [23] Australia	Cohort Study	1,220	Mean not specified, age range: 60−64	Without cognitive impairment	12 years Cognitive assessment sessions Baseline (wave-1): 2001−2002 wave-2: 2005−2006 wave-3: 2009−2010 wave-4: 2013−2014	13-MIND diet (The CSIRO-FFQ excluded butter/margarine and olive oil, so the maximum score is 13) (183-item CSIRO-FFQ, assessed intake over the past year; MIND diet score 0−13 categorized into tertiles)	Different MIND diet adherence groups: - Tertile 1: mean 5.28 - Tertile 2: mean 6.58 - Tertile 3: mean 7.82 Range: 2.5–10.5, mean = 6.3	1. Risk of dementia or Alzheimer’s disease (National Institute of Neurological Disorders criteria)
8	Mueller (2020), [40] USA	Cohort Study	828	57 ± 6.8	Without dementia, MCI and storke, Parkinson’s disease, multiple sclerosis or epilepsy	Average 6.3 years	15-MIND diet (15-item questionnaire; MIND diet score was obtained by a revised MIND questionnaire and the coding decisions that differ from the original)	To explore whether the combined influences of multiple health behaviors and multiple relevant covariates were associated with cognitive decline. Range: 3–14, mean = 9.4	1. Global cognitive function (PACC4 - RAVLT; Logical Memory II subtest from WMS-R; Digit Substitution test of the WAIS-R; MMSE) 2. Domain-specific cognitive functions (Immediate Recall; Delayed Recall; Executiive functioning)
9	Munoz-Garcia (2020), [41] Spain	Cohort Study	806	61 ± 6	Without cognitive impairment	6 years 0 (baseline), 2 years, 6 years.	15-MIND diet (136-item FFQ; MIND diet score 0−15 categorized into tertiles)	Different MIND diet adherence groups: - Tertile 1: 3.5−8 - Tertile 2: 8.5−9.5 - Tertile 3: 10−12.5	1. Domain-specific cognitive functions (STICS-m, telephone adaptation of the MMSE)
10	Dhana (2021), [42] USA	Cohort Study	569	90.8 ± 6.1	Some diagnosed with clinical AD near death, or postmortem diagnosis of AD	Not specified	15-MIND diet (144-item FFQ, assessed intake over the previous 12 months; MIND diet score 0−15)	Compare different MIND diet adherence (continuous). Mean MIND diet score: 7.35 ± 1.42	1. Global cognitive function (Episodic memory; semantic memory; working memory; perceptual speed, and visuospatial ability)
11	Melo van Lent (2021), [20] USA	Cohort Study	N = 2,092	61 ± 9	Without dementia, stroke, or other significant neurological disease	10 years Cognitive assessment: T1 (1998−2001): Baseline (n = 2092) T2 (2005–2008): average 6.6 ± 1.1 years (n = 1584) Adherence to the MIND diet score: Assessed over a maximum of 10 years	15-MIND diet (126-item FFQ, assess intake over the past 1 year; MIND diet score 0−15 categorized into tertiles)	Different MIND diet adherence groups (continuous): - Tertile 1: 1.5−5.8, median 5.0 - Tertile 2: 6.0−7.5, median 6.8 - Tertile 3: 7.7−11.7, median 8.5	1. Global cognitive function 2. Domain-specific cognitive functions (Visual Memory; Meaningful/Verbal Memory; Verbal Comprehension/Reasoning; Processing Speed; Processing Speed and Executive Function; Closure/Visual Integration and Mental Rotation)
12	Nishi (2021), [43] Spain	Cohort Study	6,647 (48% women)	65	With overweight or obesity and metabolic syndrome. Without stroke, myocardial infarction, or neurodegenerative disease	2 years Cognitive assessment: T1: Baseline T2: 2 years	15-MIND diet (143-item FFQ; MIND diet score 0−15 categorized into tertiles)	Different MIND diet adherence groups: - Lowest: 2.5–8.5, mean 8.0 - Moderate: 9.0–9.5, mean 9.0 - Highest: 10–13.5, mean 10.5	1. Global cognitive function 2. Domain-specific cognitive functions (MMSE; CDT; VFT-a and VFT-p; TMT A&B; DST-b and DST-f)
13	Boumenna (2022), [22] USA	Cohort Study	1,502	57.2 ± 7.9	40% had diabetes, 69% had hypertension, 60% had depressive symptoms	8 years Cognitive assessment: T1: Baseline T2: 2 years T3: 8 years MIND diet: T1: Baseline T2: 2 years T3: 5 years	15-MIND diet (FFQ-revised for Puerto Ricans; MIND diet score 0−15 catergorized into quintiles)	Different MIND diet adherence groups: - Quintile 1: median 5.5 - Quintile 2: median 6.5 - Quintile 3: median 7.0 - Quintile 4: median 8.0 - Quintile 5: median 9.5	1. Global cognitive function (MMSE; 16-word list learning task; digit span forward and backward; stroop test; clock drawing and figure copying; verbal fluency)
14	de Crom (2022), [21] Netherlands	Cohort Study	8,236	71.5 ± 6.9	Without dementia	Baseline I: mean = 15.6 years Baseline II: mean = 5.9 years Dememtia: Screened every 3−5 years during follow-up.	15-MIND diet (Baseline I: 170-item FFQ, assessed intake in the preceding year, interviewed by a trained dietician. Baseline II: 389-item FFQ, assessed intake in the last month, self-administered; MIND diet score 0−15 catergorized into tertiles)	Compare different MIND diet adherence groups (continuous).	1. Risk of dementia or Alzheimer’s disease (MMSE; GMS organic level; Cambridge Examination for Mental Disorders in the Elderly diagnostic interview; by consensus panel (neurologist); DSM-III-R for all-cause dementia; NINCDS-ADRDA for Alzheimer's)
15	Lotan (2022), [44] Israel	Cohort Study	960	71.6 ± 4.6	With type 2 diabetes mellitus, without dementia or any other cognitive impairment, or major neurological condition	mean = 4.1 ± 2.1 years MIND diet: Study started in 2010, FFQ was introduced in 2013. Cognitive asssessment: Assessed every 18 months, participants completed at least once.	15-MIND diet (FFQ; MIND diet score 0−15 categorized into tertiles)	Compare different MIND diet adherence groups (tertile and continuous, ranges of the tertiles are not specified).	1. Global cognitive function 2. Domain-specific cognitive functions (Episodic memory; attention/working memory; language/semantic categorization; executive function)
16	Thomas (2022), [45] France	Cohort Study	1,412	75.8 ± 4.8	Without dementia, brain tumors or major cerebrovascular pathologies	16 years median follow-up: 9.7 years range: 0.9–16.3 years	15-French MIND diet (148-item FFQ and 24-h dietary recall; French MIND diet score 0−15 categorized into tertiles)	Different MIND diet adherence groups (tertile & continuous): - Tertile 1: <6.5 - Tertile 2: 6.5–8 - Tertile 3: >8	1. Risk of dementia or Alzheimer’s disease (Neuropsychological assessments by psychologist at home; neurologist exam for diagnosis; independent committee of neurologists validate using DSM and NINCDS-ADRDA criteria)
17	Vu (2022), [46] USA	Cohort Study	8,482	70.1 - 81.8	Without dementia	MAP: 9 years, annual clinical evaluation WHIMS: 24 years, annual clinical evaluation CHAP: 18 years, clinical evaluation every 3 years.	15-MIND (FFQ; MIND diet score 0−15 categorized into tertiles)	Different MIND diet adherence groups: MAP - Tertile 1: 3.5–7.0, mean = 6.1 ± 0.8 - Tertile 2: 7.5–8.5, mean = 8.0 ± 0.4 - Tertile 3: 9.0–13.0, mean = 9.9 ± 0.9 Overall mean: 7.9 WHIIMS - Tertile 1: 2.0–5.5, mean = 4.8 ± 0.7 - Tertile 2: 6.0–7.0, mean = 6.5 ± 0.4 - Tertile 3: 7.5–12.0, mean = 8.3 ± 0.8 Overall mean: 6.7 CHAP-White - Tertile 1: 2.0–6.0, mean = 5.2 ± 0.9 - Tertile 2: 6.5–7.5, mean = 7.0 ± 0.4 - Tertile 3: 8.0–14, mean = 9.1 ± 1.1 Overall mean: 7.4 CHAP-Black - Tertile 1: 2.0–6.0, mean = 5.3 ± 0.7 - Tertile 2: 6.5–7.5, mean = 7.0 ± 0.4 - Tertile 3: 8.0–12.5, mean = 8.8 ± 0.9 Overall mean: 7.1	1. Global cognitive function - CHAP & MAP: a battery of cognitive tests; - WHIMS: 3MSE 2. Risk of dementia or Alzheimer’s disease - CHAP & MAP: Clinical evaluations for Alzheimer's and dementia in a stratified random sample; NINCDS-ADRDA criteria; - WHIMS: Cognitive tests assessed by local physician using WHIMS protocol; classified as no dementia, MCI, or probable dementia per DSM-IV; final diagnosis by WHIMS clinical panel)
18	Chen (2023), [24] USA	Cohort studies	18,136	WII: 62.2 ± 6.0 HRS: 66.5 ± 10.4 FOS: 64.2 ± 9.1	Without dementia	WII mean = 12.9 years Baseline: 2002−2004 HRS mean = 5.0 years Baseline: 2013−2018 FOS: mean = 10.7 years Baseline 1998−2001 Data were analyzed in 2022	14- and 15-MIND diet (WII lacks information on olive oil, the original score 0−14 was rescaled to 0−15) (FFQ; MIND diet score 0−15 categorized into tertiles)	Different MIND diet adherence groups: WII - Tertile 1 mean = 7 - Tertile 2 mean = 8.3 - Tertile 3 mean = 9.6 HRS - Tertile 1 mean = 5 - Tertile 2 mean = 7 - Tertile 3 mean = 9 FOS - Tertile 1 mean = 6.5 - Tertile 2 mean = 8.2 - Tertile 3 mean = 9.8	1. Risk of dementia or Alzheimer’s disease (WII: health system linkage; HRS: a validated algorithm; FOS: panel reviews for dementia identification)
19	Cornelis (2023), [47] UK	Cohort Study	120,661	Mean not specified, age ≥ 55	Without dementia	Mean = 10.5 ± 1.8 years MIND diet: Assessed every 3–4 months, completed up to four times. Cognitive assessment: Completed at least one of seven self-administered cognitive function tests.	15-MIND diet (24-h dietary recall, Oxford WebQ - a web-based tool; MIND diet score 0−15 catergorized into tertiles)	Different MIND diet adherence groups: - Tertile 1: 0−5.5 - Tertile 2: 5.5−6.5 - Tertile 3: 7.0−14.5 Mean for full sample = 6.14	1. Risk of dementia or Alzheimer’s disease (New clinical diagnosis of all-cause dementia by UKB using hospital and death records with ICD-10 codes) 2. Domain-specific cognitive functions (PM; FI; Pairs matching; RT; SDS; Trail A/Trail B)
20	Dong (2023), [48] USA	Cohort Study	1078	63.5 ± 6.7	Cognitively unimpaired, asymptomatic, with genetic risk due to a parental history of clinical AD.	Not specified Cognitive assessment: Initial follow-up four years after baseline (2001); and then follow-up every two years.	15-MIND diet (15-item self-reported diet questionnaire; MIND diet score 0−15)	Compare different MIND diet adherence (continuous). Mean MIND diet score: 9.3 ± 2.0	1. Global cognitive function (PACC; RAVLT; WMS-RLM; BVMT-R; TMT B; Stroop Neuropsychological Screening Test; WAIS-R)
21	Huang (2023), [49] China	Cohort study	4066	62.2	Without dementia	Longest 9 years Median 3 years	12-MIND diet (Excluded olive oil, butter/margarine, and cheese; replaced red wine with tea to adapt Chinese dietary culture) (3-d 24-h dietary recalls and household weighing for cooking oil and condiments.; MIND diet score 0−12 categorised into tertiles)	Different MIND diet adherence groups (tertile & continuous): - Tertile 1: 0.0−3.5, median 3.0 - Tertile 2: 4.0−4.5, median 4.0 - Tertile 3: 5.0−12.0, median 5.5 Overall median 4.5	1. Global cognitive function (TICSm) 2. Verbal memory
22	Wagner (2023), [50] USA	Cohort Study	578	84.1 ± 5.8	Without dementia	Average 9 years Cognitive assessment: Annual global cognition evaluation MIND diet: T1: 1.9 years from enrollment (70% of partcipants)	15-MIND diet (144-item FFQ, assessed intake over the past year; MIND diet score 0−15 categorized into tertiles)	Different MIND diet adherence groups: - Tertile 1: 3.5−7.0 - Tertile 2: 7.0−8.5 - Tertile 3: 8.5−12	1. Domain-specific cognitive functions (Cognitive resilience - 17 psychometric tests administered annually until death; 9 measures of post mortem neuropathology)
23	Zhang (2023), [51] UK	Cohort Study	114,684	56.8 ± 7.77	Without dementia	Average 9.4 years Cognitive assessment T1: Baseline, in 2006–2010 MIND diet 24-h dietary recall x 5, in 2009−2012 Incident dementia censoring time in the study: 29 Aug 2021	14-MIND diet (Olive oil consumption was not collected in the UK Biobank Study, so it was excluded) (24-h dietary recall, Oxford WebQ - a web-based tool; MIND diet score 14−70 categorized into tertiles)	Different MIND diet adherence groups: - Tertile 1 (lowest) - Tertile 2 (intermediate) - Tertile 3 (highest)	1. Risk of dementia or Alzheimer’s disease (All-cause dementia diagnosis from hospital records and death register data using ICD-10 and ICD-9 codes)
	**Cross-sectional studies**								
24	McEvoy (2017), [52] USA	Cross-sectional study	5,907	67.8 ± 10.8	Without dementia or AD or stroke	NA (cross-sectional study)	15-MIND diet (163-item FFQ; MIND diet score 0−15 categorized into tertiles)	Different MIND diet adherence groups: - Tertile 1: ≤6.5 - Tertile 2: 6.6–8.0 - Tertile 3: 8.1–15.0 Mean = 7.3 ± 1.8	1. Global cognition function (Immediate and delayed recall; backward counting; serial seven subtraction)
25	Calil (2018), [53] Brazil	Cross-sectional study	96	75.2 ± 6.5	36 with normal cognitive performance, 30 with MCI, 30 with AD	NA (cross-sectional study)	15-MIND diet (98-item FFQ; MIND diet score 0−15 categorized into tertiles)	Different MIND diet adherence groups: - Tertile 1 (lowest) - Tertile 2 (intermediate) - Tertile 3 (highest)	1.Domain-specific cognitive functions (MMSE; BCSB; VF; CDT; GDS)
26	Wesselman (2021), [54] Germany	Cross-sectional study	389	69 ± 6	146 with SCD, 60 with MCI, 35 with relatives of AD patients, 148 healthy	NA (cross-sectional study)	15-MIND diet (148-item FFQ; MIND diet score 0−15)	Compare different MIND diet adherence groups (continuous). (average score was 6.4 ± 1.4)	1. Domain-specific cognitive functions (Memory; language; executive function; working memory; visuospatial function)
27	Ahn (2022), [55] USA	Cross-sectional study	3,463	68.0 ± 10.0	Without dementia or history of stroke With BMI 16.5−39	NA (cross-sectional study)	15-MIND combined with high-intensity physical activity (163-item FFQ; MIND diet score 0−15 classified into two categories)	Compare different MIND diet adherence groups (± high-intensity physical activity): - Adherent: ≥ 7.5 - Non-adherent: < 7.5 The mean of participants: 7.5 ± 2.1	1. Global cognitive function (Episodic memory; Working memory; Attention/processing speed)
28	Ferreira (2022), [56] Brazil	Cross-sectional study	11,788	51.8 ± 9.01	Without previous stroke	NA (cross-sectional study)	15-MIND diet (114-item FFQ, assess intake over the past 12 months; MIND diet score 0−15)	Compare different MIND diet adherence (continuous). The mean of participants: 8.05 ± 1.54	1. Global cognition function 2. Domain-specific cognitive functions (Memory Domain; Language and Executive Function; Executive Function and Visual-spatial Organization)
29	Gauci (2022), [57] Australia	Cross-sectional study	141	52.84 ± 6.87	Without any neurological conditions, cognitive impairment, psychiatric disorders, and any health conditions that aﬀ ;ect food absorption and cognitive performance	NA (cross-sectional study)	15-MIND diet, Mediterranean Diet, DASH diet (ASA24, assessed over the past 24 h; MIND diet score 0−15)	Compare the 3 dietary patterns and different MIND diet adherence (continuous). The mean of participants: 7.06	1. Domain-specific cognitive functions (SUCCAB: Reaction and Decision Speed, Visual Processing, Spatial Working Memory, and Stroop Processing)
30	Huang (2022), [58] China	Cross-sectional study	11,245	84.06 ± 11.46	Without dementia or stroke	NA (cross-sectional study)	12-cMIND diet (The cMIND with 12 food items was developed based on the primary MIND diet to better meet the dietary characteristics of older Chinese individuals) (FFQ, investigate intake in the past one year; cMIND diet score 0−12 categorized into tertiles)	Different MIND diet adherence groups: - Tertile 1: 0–3.5, median 3.0 - Tertile 2: 4–5, median 4.5 - Tertile 3: 5.5–12, median 6 (median score was 4.5 out of 12)	1. Global cognitive function (Chinese version MMSE)
31	Lawrie (2022), [59] UK	Cross-sectional study	131	67 ± 9	Diagnosis of idiopathic Parkinson's Disease	NA (cross-sectional study)	15-MIND diet (EPIC FFQ; MIND diet score 0−15 categorized into tertiles)	Different MIND diet adherence groups (continuous): - Tertile 1: median 6.5 - Tertile 2: median 8.5 - Tertile 3: median 10.5 Range (median) across the whole sample: 4.5−12.5 (8.5)	1. Global cognitive function (MoCA adjusted for education)
32	Vassilopoulou (2022), [60] Greece	Cross-sectional study	203	70.55 ± 6.31	115 with dementia, 88 cognitively healthy	NA (cross-sectional study)	9-, 11- and 15-MIND diet (The results suggested that the 9-component MIND diet score supported the sample data better than the original 15-item MIND diet) (Certified dietitian interviewed; MIND diet score 0−15 classified into three categories)	Different 15-MIND diet adherence groups: - Low: 2.5−6.5 - Moderate: 6.5−8.5 - High: 8.5−15 Mean 9-MIND diet score (continuous) = 4.61 ± 1.56 - minimum: 0.5 - median: 4.5 - maximum: 8	1. Risk of dementia or Alzheimer’s disease (Standardized neuropsychological testing by neurologists using ICD-11; diagnosis of the neuropsychological testing of cognitive decline and dementia retrieved from medical records; grouped by cognitive status and MMSE)
33	Yeung (2022), [61] China	Cross-sectional study	3730	72.2 ± 5.0	Not specified	NA (cross-sectional study)	9-MIND diet (Insufficient information of intake of olive oil, beans, fish, poultry, fried/fast food and red meat and products) (280-item FFQ; assessed intake over the past 12 months; MIND diet score 0−9)	Compare different MIND diet adherence (continuous). Mean MIND diet score: 4.6 ± 0.9	1. Domain-specific cognitive functions (Four items from MMSE: orientation to date, orientation to address, registration of three objects, attention and calculation)
34	Escher (2023), [62] USA	Cross-sectional study	132	71.7 ± 19	Without dementia, memory problems or diagnosis, and neurological condition	NA (cross-sectional study)	15-MIND diet (15-item FFQ; MIND diet score 0−15 classified into three categories)	Different MIND diet adherence groups (continuous): - Low adherence: 0–6.5 - Moderate adherence: 7–8 - High adherence: 8.5–15	1. Domain-specific cognitive functions (Episodic memory; language functioning; executive functioning)
35	Zare (2023), [63] Iran	Cross-sectional study	60	62 ± 2	With controlled T2DM (treated with oral medications), MMSE > 24, without medical history of Parkinson’s, visual and hearing impairments	NA (cross-sectional study)	14-MIND diet (Excluded wine, which is consumed less in Muslim countries and is prohibited in Islam) (MIND dietary score questionnaire; MIND diet score 0−14 classified into three categories.)	Different MIND diet adherence groups (continuous): - Low: 0–7 - Mid: 7−14 - High: 14 98% of the participants were in mid adherence group	1. Global cognitive function (PCAP: FDST; LDMT; TMT; Stroop Task)
36	Derdiger (2024), [64] USA	Cross-sectional study	2,598	69.2 ± 0.3	Not specified	NA (cross-sectional study)	14-MIND diet (Alcohol component was dropped in MIND score to avoid penalizing non-drinkers.) (24‐h recalls; MIND diet score 0−14 categorized into tertiles)	Different MIND diet adherence groups (tertile & continuous): Tertile 1: ≤4 Tertile 2: 4.5−5.5 Tertile 3: ≥6, average 6.8 ± 0.1 (range: 0.5−11.5)	1. Global cognition function 2. Domain-specific cognitive functions (CERAD; AFT; DSST)
	**Randomised controlled trials**								
37	Arjmand (2022), [25] Iran	Randomised controlled trial	I: 22 C: 15	48 ± 5.38	Without any metabolic complication, cardiovascular diseases, Parkinson’s disease, AD, mental disorders, stroke, brain injury, cancer With BMI 30–35 kg/m2, MMSE ≥ 24	3 months Cognitive assessment: T1: Baseline T2: 3 months	Calorie-restricted modified 15-MIND diet (wine was replaced by grape, grape juice, raisins and currant) (168-item FFQ to know the usual dietary intake of ­ participants; and MIND diet score ques- tionnaire, 3-day food recall for dietary adherence; MIND diet score 0−15)	Compare the intervention group (calorie‐restricted modified MIND diet) and the control group (calorie‐restricted standard diet).	1. Domain-specific cognitive functions (FDST and BDST; LNST; SDMT; AVLT; TMT A&B; Stroop task)
38	Barnes (2023), [26] USA	Randomised controlled trial	I: 301 C: 303	70.4 ± 4.2	Without cognitive impairment With family history of dementia or AD, BMI ≥ 25, and MoCA score≥22	3 years MIND diet: Data for the key end points and covariates were collected at month 6 and then yearly until the end of the trial. Cognitive assessment: T1: Baseline T2: 6 months T3: 12 months T4: 24 months T5: 36 months	14-MIND diet with mild caloric restriction (Baseline dietary assessments: 24 -h recall, and FFQ; MIND-diet adherence: 14 items questionnaire, and individualized goal setting)	Compare the intervention group (MIND diet with mild caloric restriction) and the control group (usual diet with the same mild caloric restriction).	1. Global cognition function 2. Domain-specific cognitive functions (Episodic memory; Semantic memory; Executive function; Perceptual speed)
	**Case-control study**								
39	Filippini (2020), [65] Italy	Case-control study	EOD cases: 54 Control: 54	EOD cases: 66; Control: 64	Except EOD, not specified	NA (case-control study)	15-MIND diet (188-item FFQ, assess intake over the previous year; MIND diet score 0−15 catergorized into tertiles)	Different MIND diet adherence groups (tertile & continuous): - Tertile 1: median 6.7 - Tertile 2: median7.5 - Tertile 3: median 9.0	1. Risk of dementia or Alzheimer’s disease (Recruited EOD cases from newly-diagnosed patients at Modena Policlinico-University Hospital Memory Center and Carpi Hospital Neurology Department)

3MSE, Modified Mini-Mental State Examination; AD, Alzheimer's disease; AFT, Animal Fluency Test; AHEI, Alternative Healthy Eating Index; ASA24, Automated Self-Administered 24-Hour Dietary Assessment Tool; AVLT, Auditory Verbal Learning; BCSB, Brief Cognitive Screening Battery; BDST, Backward Digit Span Task; BVMT, Brief Visuospatial Memory Test; CDS, cognitive difficulties scale; CDT, Clock-Drawing Test; CERAD, Consortium to Establish a Registry for Alzheimer's Disease; CHAP, Chicago Health and Aging Project; CSIRO, Commonwealth Scientific and Industrial Research Organization; DASH, Dietary Approaches to Stop Hypertension; DSST, Digit Symbol Substitution Test; DSM, Diagnostic and Statistical Manual of Mental Disorders; DSM-III-R, Diagnostic and Statistical Manual of Mental Disorders 3rd Edition Revised; EBMT, East Boston Memory test; EOD, Early-onset dementia; EPIC FFQ, European Prospective Investigation into Cancer Food Frequency Questionnaire; FDST, Forward Digit Span Task; FFQ, food frequency questionnaire; FI, fluid intelligence; FOS, Framingham Heart Study Offspring cohort; FPED, Food Pattern Equivalents Database; GDS, Geriatric Depression Scale; GM, Greek Mediterranean diet; GMS, Geriatric Mental Schedule; hPDI, healthy plantbased diet index; HR, hazard ratio; HRS, Health and Retirement Study; ICD, International Classification of Diseases; LDMT, Letter Digit Modality Task; Letter F, the phonemic verbal fluency test; LNST, Letter Number Sequencing Task; MAP, Rush Memory and Aging Project; MCI, Mild Cognitive Impairment; MDP, Mediterranean dietary pattern; MDS, Mediterranean diet score; MIND, Mediterranean-DASH Intervention for Neurodegenerative delay; MMSE, Mini-Mental State Examination; MoCA, Montreal Cognitive Assessment; NC, controls with normal cognitive performance; NINCDS-ADRDA, National Institute of Neurological and Communicative Disorders and Stroke and the Alzheimer's Disease and Related Disorders Association; PACC4, Preclinical Alzheimer’s Cognitive Composite 4; PCAP, Pencil Cognitive Assessment Package; PM, prospective memory; PVD, pro-vegetarian diet; RAVLT, Rey Auditory Verbal Learning Test; RFS, recommended food score; RT, Reaction time tests; SCD, subjective cognitive decline; SDMT, Symbol Digit Modality Task; SDS, symbol digit substitution test; SMC, subjective memory complaints; STICS-m, validated Spanish Telephone Interview for Cognitive Status; SUCCAB, Swinburne University Computerized Cogntitive Assessment Battery; TICS, Telephone Interview for Cognitive Status; TICSm, Modified elephone Interview for Cognitive Status; TICSm, Telephone Interview for Cognitive Status-Modified; TMT A&B, Trail making test A and B; VF, Verbal Fluency; VFT-a and VFT-p, semantical and phonological verbal fluency tasks; WAIS-R, Wechsler Abbreviated Intelligence Scale-Revised; WHIMS-ECHO, WHIMS-Epidemiology of Cognitive Health Outcomes; WHIMS, Women’s Health Initiative Memory Study; WII, Whitehall II study; WL-CERAD, the word list from the Consortium to Establish a Registry for Alzheimer’s Disease; WMS-R, Wechsler Memory Scale—Revised; WMS-RLM, Wechsler Memory Scale–Revised Logical Memory subtest.

* “continuous” signifies that the authors treated MIND diet adherence scores as continuous variables, analyzing overall adherence levels from low to high. When “tertile and continuous" are indicated, it means comparisons were made both across overall adherence levels and specific differences between the categorized groups.

# Please refer to Appendix III for details on calculating global cognitive scores and administering domain-specific cognitive tests.

The assessment of the MIND diet is not uniform across all studies. In the majority of the studies (n = 29/39, 74.4%), Food Frequency Questionnaires (FFQs) were employed to evaluate the dietary patterns and adherence of participants. This method requires participants to recall their dietary intake over periods ranging from 1 month to 12 months. One study supplemented the FFQ with a 24 -h dietary recall, weight assessment, and individualized goals, among other measures; another supplemented with household weighing for cooking oil and condiments. A subset of studies (n = 3/39, 7.7%) relied on web-based 24-h dietary record tools to assess adherence to the MIND diet. One study used the Automated Self-Administered 24-Hour Dietary Assessment Tool to measure dietary intake. Only one study specified that they utilized certified dietitians to conduct interviews and evaluate dietary patterns and adherence. Most studies adhere to the same MIND diet scoring system, which assigns scores to 15 components (n = 29/39, 74.4%), and many categorized MIND diet adherence scores into tertiles or quintiles. However, the cut-off scores for high, moderate, and low adherence to the MIND diet varied across the studies. Moreover, several studies made slight adjustments to the MIND diet for specific reasons, resulting in scoring modifications ranging from 9 to 14 components (n = 10/39, 28.2%). These adjustments were made to account for variations in dietary assessment tools, cultural and dietary practices, and the availability of specific food items. Consequently, the total MIND score in these studies was adapted to reflect these modifications. The methods and measures used to assess cognitive health were also highly heterogeneous. Detailed reasons behind these adjustments are listed in the table.

Details on how the risk of dementia and Alzheimer’s disease was assessed, along with information on the diagnostic criteria used, are listed in the “Outcome” column of Table 1. This section also includes a brief description of the outcome measures for global cognitive function and domain-specific cognitive functions. Comprehensive details on the methods for calculating global cognitive scores and administering domain-specific cognitive tests are provided in Appendix III.

### 3.3 Risk of bias in studies

The results of the studies’ critical appraisal and risk of bias, organized by study types, are illustrated in Table 2. Almost all the cohort studies (n = 21/23, 91.3%) were classified as good quality. The quality of the cross-sectional studies was good (n = 10/13, 76.9%). All RCTs were of good quality (n = 2/2, 100%). However, the only case-control study was classified as poor quality (n = 1/1, 100%).

**Table 2:** Results of critical appraisal using JBI scoring.

Author(s)	Title	JBI score	Quality
**Cohort studies**			
Morris (2015)	MIND diet associated with reduced incidence of Alzheimer's disease	9/11	Good
Morris (2015)	MIND diet slows cognitive decline with aging	10/11	Good
Berendsen (2018)	Association of Long-Term Adherence to the MIND Diet with Cognitive Function and Cognitive Decline in American Women	8/11	Good
Shakersain (2018)	The Nordic prudent diet reduces risk of cognitive decline in the Swedish older adults: a population-based cohort study	6/11	Poor
Adjibade (2019)	Prospective association between adherence to the MIND diet and subjective memory complaints in the French NutriNet-Santé cohort	8/11	Good
Cherian (2019)	Mediterranean-Dash Intervention for Neurodegenerative Delay (MIND) Diet Slows Cognitive Decline After Stroke	9/11	Good
Hosking (2019)	MIND not Mediterranean diet related to 12-year incidence of cognitive impairment in an Australian longitudinal cohort study	11/11	Good
Mueller (2019)	Self-reported health behaviors and longitudinal cognitive performance in late middle age: Results from the Wisconsin Registry for Alzheimer's Prevention	9/11	Good
Munoz-Garcia (2020)	"A priori" Dietary Patterns and Cognitive Function in the SUN Project	8/11	Good
Dhana (2021)	MIND diet, common brain pathologies, and cognition in community-dwelling older adults	6/11	Poor
Melo van Lent (2021)	Mind Diet Adherence and Cognitive Performance in the Framingham Heart Study	8/11	Good
Nishi (2021)	Mediterranean, DASH, and MIND Dietary Patterns and Cognitive Function: The 2-Year Longitudinal Changes in an Older Spanish Cohort	9/11	Good
Boumenna (2022)	MIND Diet and Cognitive Function in Puerto Rican Older Adults	8/11	Good
de Crom (2022)	MIND diet and the risk of dementia: a population-based study	9/11	Good
Lotan (2022)	Greater intake of the MEDI diet is associated with better cognitive trajectory in older adults with type 2 diabetes	9/11	Good
Thomas (2022)	Association of a MIND Diet with Brain Structure and Dementia in a French Population	8/11	Good
Vu (2022)	Adherence to MIND Diet, Genetic Susceptibility, and Incident Dementia in Three US Cohorts	8/11	Good
Chen (2023)	Association of the Mediterranean Dietary Approaches to Stop Hypertension Intervention for Neurodegenerative Delay (MIND) Diet With the Risk of Dementia	9/11	Good
Cornelis (2023)	MIND Dietary Pattern and Its Association with Cognition and Incident Dementia in the UK Biobank	9/11	Good
Dong (2023)	Identification of plasma metabolites associated with modifiable risk factors and endophenotypes reflecting Alzheimer’s disease pathology	8/11	Good
Huang (2023)	Mediterranean-dietary approaches to stop hypertension intervention for neurodegenerative delay (MIND) diet and cognitive function and its decline: a prospective study and meta-analysis of cohort studies	9/11	Good
Wagner (2023)	The association of MIND diet with cognitive resilience to neuropathologies	9/11	Good
Zhang (2023)	Associations of Midlife Dietary Patterns with Incident Dementia and Brain Structure: Findings from the UK Biobank Study	7/11	Good

**Cross-sectional Studies**			
McEvoy (2017)	Neuroprotective Diets Are Associated with Better Cognitive Function: The Health and Retirement Study	8/8	Good
Calil (2018)	Adherence to the Mediterranean and MIND diets is associated with better cognition in healthy seniors but not in MCI or AD	5/8	Good
Wesselman (2021)	Dietary patterns are related to cognitive functioning in elderly enriched with individuals at increased risk for Alzheimer’s disease	7/8	Good
Ahn (2022)	Association of adherence to high-intensity physical activity and the Mediterranean-dietary approaches to stop hypertension intervention for neurodegenerative delay diet with cognition: A cross-sectional study	8/8	Good
Ferreira (2022)	Association Between Adherence to the MIND Diet and Cognitive Performance is Affected by Income: The ELSA-Brasil Study	4/8	Poor
Gauci (2022)	The Association Between Diet and Cardio-Metabolic Risk on Cognitive Performance: A Cross-Sectional Study of Middle-Aged Australian Adults.	8/8	Good
Huang (2022)	Development of the cMIND Diet and Its Association with Cognitive Impairment in Older Chinese People	6/8	Good
Lawrie (2022)	Dietary patterns and nonmotor symptoms in Parkinson’s disease: a cross-sectional analysis	5/8	Good
Vassilopoulou (2022)	Adjustment of the MIND diet tool for discriminating Greek patients with dementia: A confirmatory factor analysis	3/8	Poor
Yeung (2022)	Dietary patterns and intrinsic capacity in community-dwelling older adults: a cross-sectional study	6/8	Good
Escher (2023)	Roles of physical activity and diet in cognitive aging: is more better?	6/8	Good
Zare (2023)	Adherence to Mediterranean-Dash Intervention for Neurodegenerative Delay (MIND) dietary pattern in elderly with type 2 diabetes and the correlation with cognitive functions and metabolic profile	4/8	Poor
Derdiger (2024)	Cognitive performance in relation to MIND and MEPA III dietary pattern accordance of NHANES participants	8/8	Good

**Randomized Controlled Trials**			
Arjmand (2022)	Effect of MIND diet intervention on cognitive performance and brain structure in healthy obese women: a randomized controlled trial	8/13	Good
Barnes (2023)	Trial of the MIND Diet for Prevention of Cognitive Decline in Older Persons	10/13	Good

**Case-control Studies**			
Filippini (2020)	Dietary Habits and Risk of Early-Onset Dementia in an Italian Case-Control Study	3/10	Poor

*Note: Cut-off JBI scores: Cohort study - 7 out of 11; Cross-sectional study - 5 out of 8; Randomized controlled trial - 8 out of 13; Case-control study - 6 out of 10.

### 3.4 Result of synthesis

In this study, we synthesize data extracted from the selected articles by grouping the outcome measures into three key cognitive health outcomes. These measures include 1) global cognitive function, 2) the risk of dementia and Alzheimer’s disease, and 3) domain-specific cognitive functions. A concise table categorized by outcome and then sequenced by study design is included in Table 3 to facilitate quick reference.

**Table 3:** Results of individual studies.

First author (year), country	Study design	Population size	Result	Significance of result (p-value)
**Global cognitive function**				
Morris (2015), USA	Cohort study	N = 960	+ve	p < 0.0001
Berendsen (2018), USA	Cohort study	N = 16058	no significant association	p = 0.88
Shakersain (2018), Sweden	Cohort Study	N = 2223	+ve	p = 0.019 (moderate adherence) p < 0.001 (high adherence)
Cherian (2019), USA	Cohort study	N = 106	+ve	p = 0.034
Dhana (2021), USA	Cohort Study	N = 569	+ve	p = 0.003 (better cognition) p = 0.005 (better cognition independent of overall brain pathologies)
Melo van Lent (2021), USA	Cohort study	N = 2,092	+ve	p = 0.004
Nishi (2021), Spain	Cohort study	N = 6,647	no significant association	NA
Boumenna (2022), USA	Cohort study	N = 1,502	+ve	p = 0.0019
Huang (2022), China	Cross-sectional study	N = 11,245	+ve	p < 0.001
Lotan (2022), Israel	Cohort Study	N = 960	+ve	p = 0.043
Dong (2023), USA	Cohort Study	N = 1078	+ve	p = 0.012 (Mediation effects on PACC)
Huang (2023), China	Cohort study	N = 4066	+ve	p < 0.001
McEvoy (2017), USA	Cross-sectional study	N = 5,907	+ve	p < 0.001
Ferreira (2022), Brazil	Cross-sectional study	N = 11,788	+ve	p = 0.03
Ahn (2022), USA	Cross-sectional study	N = 3,463	+ve	p < 0.001
Lawrie (2022), UK	Cross-sectional study	N = 131	no significant association	p = 0.054
Zare (2023), Iran	Cross-sectional study	N = 60	no significant association	p > 0.05
Derdiger (2024), USA	Cross-sectional study	N = 2,598	+ve	p < 0.001
Barnes (2023), USA	RCT	Intervention: N = 301 Control: N = 303	no significant association	p = 0.23

**The risk of dementia and Alzheimer’s disease**				
Morris (2015), USA	Cohort study	N = 923	+ve	p = 0.0006
Adjibade (2019), France	Cohort study	N = 6,011	+ve	p = 0.03
Hosking (2019), Australia	Cohort study	N = 1,220	+ve	p = 0.004
Vu (2022), USA	Cohort study	MAP: N = 725 WHIMS: N = 5,308 CHAP: N = 2,449	MAP & WHIMS: +ve CHAP: no significant association	p < 0.0001
Thomas (2022), France	Cohort study	N = 1,412	+ve	p = 0.003 (dementia); p = 0.008 (AD)
de Crom (2022), Netherlands	Cohort study	Baseline I: N = 5,375 Baseline II: N = 2,861	+ve	p = 0.005 (baseline II)
Zhang (2023), UK	Cohort study	N = 114,684	+ve	p = 0.115
Chen (2023), USA	Cohort study	WII: N = 8,358 HRS: N = 6,758 FOS: N = 3,020	+ve	p = 0.01
Cornelis (2023), UK	Cohort study	N = 120,661	+ve among females; no significant association among males	p = 0.008 (among females); p = 0.11 (among males)
Vassilopoulou (2022), Greece	Cross-sectional study	N = 203	+ve	p < 0.001
Filippini (2020), Italy	Case-control study	EOD cases: N = 54 Control: N = 54	+ve	NA

**Domain-specific cognitive functions**				
Morris (2015), USA	Cohort study	N = 960	+ve	p < 0.0001 (semantic memory; perceptual speed) p = 0.001 (episodic memory)
Berendsen (2018), USA	Cohort study	N = 16058	+ve	p < 0.0001 (verbal memory)
Cherian (2019), USA	Cohort study	N = 106	+ve	p = 0.043 (semantic memory) p = 0.059 (perceptual speed)
Mueller (2020), USA	Cohort study	N = 828	+ve	p = 0.027 (executive function)
Munoz-Garcia (2020), Spain	Cohort study	N = 806	+ve	p = 0.09 (STICS-m)
Melo van Lent (2021), USA	Cohort study	N = 2,092	+ve	p = 0.01 (Visual Memory); p = 0.01 (Processing Speed)
Nishi (2021), Spain	Cohort study	N = 6,647	+ve	p = 0.045 (working memory)
Lotan (2022), Israel	Cohort study	N = 960	+ve	p = 0.015 (executive functions)
Wagner (2023), USA	Cohort study	N = 578	+ve	p = 0.001 (cognitive resilience)
Calil (2018), Brazil	Cross-sectional study	N = 96	+ve	p = 0.007 (MMSE); p = 0.014 (learning scores)
Wesselman (2021), Germany	Cross-sectional study	N = 389	+ve	p = 0.029 (memory)
Ferreira (2022), Brazil	Cross-sectional study	N = 11,788	+ve	P < 0.001 (executive function); P = 0.025 (better executive function among those with high income)
Gauci (2022), Australia	Cross-sectional study	N = 141	+ve	p = 0.035 (executive functioning)
Yeung (2022), China	Cross-sectional study	N = 3730	no significant association	p = 0.032 (men) p = 0.010 (women)
Escher (2023), USA	Cross-sectional study	N = 132	+ve	p = 0.14 (episodic memory) p = 0.09 (executive functions) p = 0.06 (language)
Derdiger (2024), USA	Cross-sectional study	N = 2,598	+ve	p < 0.001 (in all cognitive tests)
Arjmand (2022), Iran	RCT	Intervention: N = 22 Control: N = 15	+ve	p < 0.05 (working memory, verbal recognition memory, and attention)
Barnes (2023), USA	RCT	Intervention: N = 301 Control: N = 303	no significant association	p = 0.23 (episodic memory, semantic memory, executive function, and perceptual speed)

AD, Alzheimer's disease; CHAP, Chicago Health and Aging Project; EOD, Early-onset dementia; FOS, Framingham Heart Study Offspring cohort; HRS, Health and Retirement Study; MAP, Rush Memory and Aging Project; MMSE, Mini-Mental State Examination; NA, Not Available; PACC, Preclinical Alzheimer’s Cognitive Composite; STICS-m, validated Spanish Telephone Interview for Cognitive Status; WHIMS, Women’s Health Initiative Memory Study; WII, Whitehall II study; +ve, Positive correlation between MIND diet adherence and cognitive health.

#### 3.4.1 Global cognitive function

Nineteen articles examined the association between MIND diet adherence and global cognitive function. These included cohort studies (n = 11/19, 57.9%), cross-sectional studies (n = 7/19, 36.8%), and RCT (n = 1/19, 5.3%). The majority of the studies (n = 14/19, 73.7%) demonstrated significantly favourable results, while the remaining five did not show a significant association.

Among the fourteen studies with favourable outcomes, nine were cohort studies. One study found that higher adherence to the MIND diet was linked to slower cognitive decline in global cognition (p = 0.034), [39] while another noted that higher MIND diet scores were associated with improved global cognitive function (p = 0.004) [20]. A third study confirmed these findings, associating higher diet quintiles with better global cognition (p = 0.0019) [22]. The MIND diet score significantly correlated with slower cognitive decline (p < 0.0001).^11^ Moderate (p = 0.019) and high adherence (p < 0.001) were linked to less decline in MMSE scores. [37] Additionally, a higher MIND diet score was tied to improved cognitive function (p = 0.003) and performance, even with brain pathologies (p = 0.005) [42]. Another study indicated that the MIND diet slowed declines in global cognition (p = 0.043) [44]. Mediation by beta-cryptoxanthin influenced the relationship between the MIND diet and the PACC (p = 0.012) [48]. Higher MIND diet scores were also linked to better verbal memory and global cognitive function (p < 0.001). [49].

Five cross-sectional studies yielded positive results. One study demonstrated that higher adherence to the MIND diet was significantly associated with better global cognitive scores (p < 0.001). [52] Another study supported this, showing that higher MIND diet scores were associated with better global cognitive performance across all cognitive tests (p < 0.001). [64]. In a third study, which included physical activity as an additional intervention, significant differences in cognitive scores were observed between groups. When the MIND diet was the sole intervention, higher adherence was associated with better global cognition (p < 0.001). [55]. A fourth cross-sectional study found a significant association between MIND diet adherence and global cognition, but only when income was considered (p = 0.03) [56]. Lasly, it was found that individuals in the highest tertile of cMIND diet adherence, which aligns with dietary characteristics of older Chinese individuals, had a lower risk of cognitive impairment compared to those in the lowest tertile (p < 0.001). [58].

The five remaining studies, including two cohort studies, two cross-sectional studies, and one RCT, did not find a significant association between the MIND diet and cognitive function. One cohort study reported no significant beneficial associations between cognitive performance and the MIND diet. [43] Another cohort study concluded that greater MIND diet adherence was not linked to better global cognition (p-trend = 0.88) [19]. The only RCT included in this review showed that improvements in global cognition scores from baseline to year 3 did not differ significantly between the trial groups, with an estimated mean difference of 0.035 standardized units (p = 0.23).^26^ Additionally, a UK study found no significant associations between the MIND diet and cognitive function (p = 0.054) when measured by the MoCA, adjusted for education, among participants with idiopathic Parkinson's disease. [59] Furthermore, An Iran study showed that the level of adherence to the MIND dietary pattern did not significantly correlate with cognitive functions (p > 0.05). [63].

#### 3.4.2 The risk of dementia and Alzheimer’s disease

Eleven studies examined the association between the risk of dementia and Alzheimer’s disease with MIND diet adherence. These included cohort studies (n = 9/11, 81.8%), a cross-sectional study (n = 1/11, 9.1%), and one case-control study (n = 1/11, 9.1%). A vast majority of them showed positive results (n = 10/11, 90.9%).

A vast majority of cohort studies (n = 8/9, 88.9%) reported positive results. One cohort study demonstrated that individuals in the second and highest tertiles of MIND diet scores had lower rates of Alzheimer's disease compared to those in the lowest tertile (p = 0.0006). [17] Another cohort study, which included three study populations, found that in two of these populations, individuals in the highest tertile of MIND adherence had a significantly lower risk of all-cause dementia compared to those in the lowest tertile (p < 0.0001). [46]. Another study observed that the MIND diet reduced the odds of dementia incidence (p = 0.004) when comparing different dietary patterns and MIND adherence, and this association persisted only for the MIND diet when more demographic and lifestyle variables were included (p = 0.016) [23].

Similar findings were reported in two other cohort studies, where each 1-point increase in the French MIND diet score was associated with a lower risk of both all-cause dementia (p = 0.003) and AD (p = 0.008), [45] and higher MIND diet scores were associated with a lower risk of dementia (p = 0.01) [24]. Another two cohort studies also showed positive results, but the level of significance was not specified. One of these studies indicated that over a mean follow-up period of 5.9 years, a higher MIND diet score at baseline was associated with a lower risk of dementia in each follow-up interval (p = 0.005), although the strength of these associations slightly decreased over time. [21] Another study found that the MIND diet demonstrated protective effects against the onset of dementia, although the association was not statistically significant (p = 0.115); and in subgroup analysis, higher MIND adherence was significantly associated with a lower incidence risk of dementia among females in the study sample [51].

One study investigated subjective memory complaints (SMC) as the outcome measure, which is considered a possible precursor of AD. This study did not show significant results among the full sample and participants aged 60–69 years (p-trend = 0.11 and 0.39, respectively). However, a significant inverse association was observed between MIND adherence and SMC among participants aged ≥70 years (p = 0.03). [38] Only one cohort study investigating the association between MIND adherence and incident dementia did not show significant results overall. This study found an inverse association among females (p = 0.008) but not among males (p = 0.11) [47].

The cross-sectional study conducted in Greece utilised the MIND-9 to measure diet adherence, which is a validated tool considered suitable for evaluating diet adherence in the Greek population. The results indicated that MIND-9 accounted for significant variance in MMSE scores (p < 0.001), and the dementia group exhibited lower MIND adherence compared to the healthy group. [60]

The case-control study examined the association between dietary factors and the risk of early-onset dementia (EOD). It was found that the control group had a higher mean MIND diet adherence score compared to the case group. Risk analysis based on tertile distribution revealed a lower risk of EOD with increased adherence to the MIND diet, particularly in the highest adherence tertile. [65]

#### 3.4.3 Domain-specific cognitive functions

Among the eighteen articles investigating the effect of the MIND diet and its adherence on domain-specific cognitive functions, nearly all, including nine cohort studies, six cross-sectional studies, and one RCT, showed positive results. Only one cross-sectional study and one RCT showed no significant association.

Of the nine included cohort studies showing positive results, most of them are of high significance. One cohort showed that greater long-term adherence to the MIND score was related to significantly higher verbal memory scores (p < 0.0001). [19] Another utilized cognitive resilience (CR) as the outcome measure, which is calculated using longitudinal data on global cognition and nine measures of post-mortem neuropathology. It showed that compared to individuals in the lowest tertile of the MIND score, those in the top tertile had higher cognition (p = 0.001) [50]. One cohort showed that higher MIND adherence was associated with better visual memory (p = 0.01), processing speed (p = 0.01), etc [20]. Another showed that adherence to the MIND diet was associated with the backward recall Digit Span Test assessment of working memory (p = 0.045) [43]. Comparing the top and the lowest tertile of MIND diet scores, the result showed that MIND diet was associated with a slower decline in semantic memory (p = 0.043) and perceptual speed (p = 0.059) [39]. The MIND diet score was also found to be associated with a slower decline in executive function (p = 0.027) [40]. Another cohort measuring cognitive outcome by STICS-m (telephone adaptation of the MMSE) showed a positive association between the MIND diet and cognitive function that each 1-SD increase in the MIND increased the 6-year change in STICS-m score by 0.27 points (p = 0.09) [41]. The MIND diet score was positively and significantly related to each cognitive domain, particularly episodic memory (p = 0.001), semantic memory (p < 0.0001), and perceptual speed (p < 0.0001). [11]. Additionally, higher intake of the MIND diets was associated with a slower rate of decline in executive functions (p = 0.015) after adjusting for socio-demographic factors. [44]

In the seven cross-sectional studies, the outcome measures varied and showed beneficial associations with the MIND diet. Six of them reported positive results. A study showed that MIND diet scores were associated with better performance on all cognitive tests (p < 0.001), they assessed cognition, including immediate learning and delayed recall of new information, and the categorical verbal fluency domain of executive function. [64] Similarly, another study conducted a neuropsychiatric battery, including the Mini-Mental State Examination, significant associations were found between the MIND diet adherence and the MMSE (p = 0.007), and higher learning scores were found among those in the highest tertile of the MIND diet adherence score (p = 0.014) [53]. Other studies also showed that adherence to the MIND diet was significantly related to executive functioning, the Stroop processing domain (p = 0.035) [57], and a greater MIND diet score was associated with better memory (p = 0.029) [54]. One study also showed a significant association when including income in the comparison. MIND diet adherence was associated with executive function (p < 0.001), and for participants with high income, greater adherence was associated with better executive function (p = 0.025). [56]. Another study indicated that higher adherence to the MIND diet was associated with improved cognitive outcomes, including episodic memory (p = 0.14), language (p = 0.06), and executive functions (p = 0.09), particularly when physical activity levels were low [62].In contrast, only one cross-sectional study found no significant associations between higher MIND diet adherence and the likelihood of greater cognitive domain scores in both men (p = 0.032) and women (p = 0.010) [61].

The one RCT showing positive results used a diverse battery of neurocognitive tests to measure cognitive performance in different domains. The results found in the MIND diet group had better improvement in working memory, verbal recognition memory, and attention compared with the control group (p < 0.05). [25] The other RCT included in this review showed that the changes in different cognition domains, including episodic memory, semantic memory, executive function, and perceptual speed from baseline to year 3, did not differ significantly (p = 0.23) [26].

## 4 Discussion

This systematic review examined the link between the MIND diet and cognitive health, focusing on cognitive degenerative diseases and functions in middle-aged and older adults. Of the studies, 14 out of 19 articles explored MIND diet adherence and global cognitive function, showing positive results. 10 out of 11 studies investigated MIND diet adherence and dementia/Alzheimer’s risk, showing positive associations. 16 out of 18 articles examined the MIND diet's effect on domain-specific cognitive functions, with favorable outcomes. Our review synthesizes these studies, offering insights into the MIND diet's impact on global cognitive function, Alzheimer’s/dementia risk, and domain-specific cognitive functions. The key findings are fivefold.

Firstly, all observational studies reviewed consistently demonstrate positive outcomes from adherence to the MIND diet, suggesting its beneficial role in enhancing global cognitive function. These align with evidence supporting that dietary patterns rich in brain-healthy nutrients can mitigate cognitive decline. [17] A systematic review indicates that adherence to the MIND diet is associated with improved cognitive performance and lower risk of cognitive decline in older adults [66]. This may help reduce Alzheimer's/dementia risk through various biological mechanisms. For instance, limiting saturated and trans fats can enhance blood-brain barrier function and decrease amyloid-beta (Aβ) accumulation, a key factor in neurodegenerative diseases [66]. The MIND diet emphasizes intake of 10 brain-healthy foods, which provide essential nutrients and antioxidants that protect brain cells and reduce inflammation, a key factor in cognitive performance [11,[17,67,68]. Clinicians should incorporate the MIND diet into guidelines for preventing Alzheimer’s and dementia, especially among middle-aged and older adults at higher risk. Future research should further validate these findings and explore the diet's effectiveness across different populations.

Secondly, the two RCTs examined produced conflicting results, with one showing a positive association [25] and the other indicating no significant impact of the MIND diet [26]. Both studies recruited obese populations and implemented calorie-restricted diets. Additionally, the RCT showing no significant difference included participants with a family history of AD/dementia, a crucial risk factor [69,70]. Given that dietary fat type significantly affects dementia risk, a higher intake of saturated fats and trans fats is associated with an increased Alzheimer's risk [71], calorie restriction might confer cognitive benefits, potentially confounding results. Future research should conduct RCTs in non-obese populations to isolate the MIND diet’s specific benefits from those of calorie restriction.

Thirdly, the domain-specific results reveal that the MIND diet particularly benefits memory and executive function, with 16 out of 18 papers demonstrating significant positive results. The most consistent evidence was found for different types of memory, including semantic, episodic, verbal, visual, and working memory (n = 10), [11,19,20,25,26,39,43,54,62,64] followed by executive functions (n = 7).^26^,^40^,^44^,^56^,^57^,^62^,^64^] Adhering to the MIND diet could serve as an effective dietary intervention for enhancing cognitive health. Clinicians can use this information to advocate for dietary changes to support cognitive health, particularly for middle-aged and older adults at risk of cognitive decline. Nutrients in the MIND diet, such as antioxidants, omega-3 fatty acids, and vitamins, reduce oxidative stress and promote neuroplasticity.^72^] As a result, the MIND diet has been reported as beneficially related to frailty components and functioning parameters [73]. It also incorporates foods rich in flavonoids and vitamin E, which inhibit Aβ formation [68], whose accumulation in the brain is associated with the development of Alzheimer's disease, cognitive decline, and neurodegeneration. The consumption of long-chain n-3 fatty acids found in fish also contributes to cognitive health by reducing oxidative damage. [74] These mechanisms explain why the MIND diet can improve specific cognitive domains. If validated by evidence and RCTs, it could become a cornerstone of public health strategies to improve cognitive health and promote healthy aging. Policymakers and health educators could leverage these findings to advocate for MIND-centered dietary interventions that enhance memory and executive function in aging populations [75]. Furthermore, public health policymakers should emphasize these dietary patterns among younger populations through nutrition education on the benefits of the MIND diet.

Of the 39 studies analyzed, 18 categorized MIND diet adherence scores into tertiles, finding significant positive effects on cognitive health in the 2nd and 3rd tertiles, though two studies lacked specified p-values for comparisons. Our critical appraisal using JBI scoring found 16 of these 18 studies to be of good quality. The Tertile 2 score range varied widely, from unspecified values to specific scores such as 4.0–5.0 (for cMIND total 12), [58] 4.5−5.5 (for MIND total 14) [64], 5.5−6.5 (for MIND total 15) [38,47], 8.5−9.5 (for MIND total 15) [41], and 9.0−9.5 (for MIND total 15) [43]. Besides, 17 studies examine MIND diet adherence as continuous variables, while some categorized scores into quintiles, revealing significant positive effects from Quintile 4 (MIND diet score 6.7−7.3) and Quintile 5 (7.4−11.0), [19] or significant positive effects were observed from Quintile 2 (median score of 6.5) to Quintile 5 (median score of 9.5).^22^ These findings underscore the effectiveness of categorizing participants by MIND diet adherence when comparing cognitive assessment results. Our systematic review provides rich information on the characteristics of the included studies. It highlights that future research on the MIND diet and cognitive health could benefit from reference studies with similar age ranges or demographics. This will facilitate meaningful categorizations of MIND diet adherence groups and enhance the accuracy of comparative analyses across populations.

Lastly, of the 10 studies not specifying the use of a Food Frequency Questionnaire (FFQ), four actually employed FFQs that applied the MIND scoring. [26,40,48,63] While these FFQs may differ, the scoring system remains consistent. The remaining studies employed 24 -h recall methods (n = 6) [38,47,49,51,57,64], with three using a web-based record tool, [38,47,51] including the Oxford WebQ,^47^,^51^] while the fourth referenced a validated web-based 24 -h dietary record tool for self-administration without specifications. [38] Adherence measured by these alternative methods significantly classified cognitive outcomes, while relationships were also observed between cognitive outcomes and the MIND diet adherence as measured traditionally by the FFQ. This provides indirect evidence for the convergent validity of the MIND questionnaire. Therefore, using shorter screening tools, such as the MIND diet questionnaire, is effective for classifying adherence. Clinicians should consider adopting these time-efficient instruments in clinical practice.

### 4.1 Strengths

Meta-analysis is appropriate only when the combined studies are sufficiently similar in participants, interventions, and outcomes, ensuring that a meaningful. [76] Due to methodological variations, such as differences in outcome definitions and measurements, we used alternative synthesis methods to account for the heterogeneity among included studies. A key strength of this review is its adherence to PRISMA guidelines [30] and SWiM extension [31]. These frameworks ensure a transparent and systematic approach to data synthesis. The Cochrane Handbook for Systematic Reviews of Interventions also suggests that although alternative synthesis methods yield results that are more limited for decision-making compared to meta-analysis, the PRISMA and SWiM guidelines can be additional assistance to authors when the required data for meta-analysis are not available [77]. Additionally, our review includes middle-aged participants, broadening the understanding of the MIND diet's impact beyond the eldely. This review specifically focuses on clinical outcomes related to cognitive health and dementia risk, enhancing knowledge of the MIND diet's role in mitigating cognitive decline.

Another strength is our comprehensive search strategy, including hand searches for publications between April 2023 and March 2024, which brought in new articles not included in prior reviews, thereby providing a more up-to-date perspective on the MIND diet's impact. By categorizing cognitive health outcomes into three key areas, we systematically assess the effects of the MIND diet, offering a more detailed and holistic perspective that is highly applicable to clinical practice and future research.

### 4.2 Limitations

However, our review has limitations. First, the heterogeneity in the study population, intervention, outcome measurements, and designs posed significant challenges, limiting our ability to conduct a meta-analysis and necessitated SWiM guidelines. Additionally, many studies did not consistently report health status or covariates such as age, sex, ApoE4 status, BMI. This lack of detail, along with diverse statistical data, prevented a meta-analysis or subgroup analysis.

Furthermore, methodological quality varied, affecting reliability. Some studies had small sample sizes or short follow-up periods, limiting the generalizability. Methodological differences in dietary assessment tools significantly influence findings on the MIND diet's impact on cognitive health. Comprehensive FFQ capture dietary intake more accurately, while shorter FFQs might miss components. Shorter recall periods yield more accurate reporting, whereas longer periods can introduce bias. Variability in measuring cognitive outcomes complicates direct comparisons and may introduce inconsistencies. Standardizing these methods in future research could enhance comparability clarify the relationship between the MIND diet and cognitive health.

Variations in scoring systems (e.g., 0−9, 0−12, 0−15) can influence participant categorization, while grouping methods (e.g., tertiles vs. quintiles) and varied can impact statistical power and result interpretation. These differences lead to inconsistencies in findings and challenge definitive conclusions. Most studies categorized participants by MIND diet adherence into tertiles or quartiles, or treated adherence as a continuous score, but the cut points varied, complicating the definition of an optimal minimum MIND diet score for cognitive health improvement. Further research is essential to establish a clinically meaningful MIND diet score that promotes cognitive health effectively.

Another limitation is the potential for publication bias. Despite our comprehensive search strategy, some relevant studies, especially those with negative results, may not have been identified. Published literature often favors studies with significant findings, leading to overrepresentation of positive results and underrepresentation of null findings. [78] Publication bias is a well-known issue that can distort the overall understanding of the MIND diet’s effects.

Lastly, although our review includes middle-aged participants, most studies focused on older adults, which might impact the generalizability of our findings to a wider age range.

## 5 Conclusion

In conclusion, this systematic review emphasizes the MIND diet’s significant potential for improving cognitive health, particularly in enhancing global cognitive function, memory, and executive function. Observational studies consistently support incorporating the MIND diet into clinical guidelines for the prevention and management of Alzheimer’s disease and dementia among middle-aged and older adults at high risk. However, mixed results from randomized controlled trials indicate a pressing need for further research, especially in non-obese populations, to isolate the specific benefits of the MIND diet distinct from calorie restriction. Our review, conducted with methodological rigor in adherence to PRISMA and SWiM guidelines, offers reliable and valid insights by focusing on clinical outcomes related to cognitive health and dementia risk. By categorizing cognitive health outcomes into three key areas, we present a comprehensive perspective valuable for clinical practice and future research. Additionally, the practical application of MIND diet adherence assessments via alternative methods, such as MIND diet questionnaires, demonstrates its effectiveness in classifying MIND diet adherence and differentiating cognitive outcomes. Despite these strengths, our review acknowledges limitations, such as the diversity in study populations, interventions, and study designs, which impact the consistency and reliability of synthesized findings. Discrepancies in scoring systems and grouping methods further complicate defining a clear minimum or optimal MIND diet score for cognitive enhancement. These limitations highlight the necessity for further research focused on standardizing assessment criteria to establish definitive recommendations for the MIND diet's role in promoting cognitive health across diverse populations.

## ORCID ID

Jenny Hiu Wai Tse: 0000-0002-1042-0768

Queenie Pui Sze Law: 0000-0002-1388-0372

Jenny Tsun Yee Tsang: 0000-0003-4558-9384

Lorna Kwai Ping Suen: 0000-0002-0126-6674

Stefanos Tyrovolas: 0000-0003-4797-7743

Rick Yiu Cho Kwan: 0000-0002-4332-780X

## Registration and protocol

The review protocol has been registered at PROSPERO (Identifier: CRD42023391972). The latest version of the protocol was published online at https://www.crd.york.ac.uk/prospero/display_record.php?ID=CRD42023391972 on 2nd February 2023.

## Support

Nil.

## Funding

Nil.

## Availability of data

The datasets during and/or analyzed during the current study are available from the corresponding author upon reasonable request.

## Declaration of competing interest

The authors declare that they have no competing interests.

## References

[1] Bishop N.A., Lu T., Yankner B.A. (2010 Mar 25). Neural mechanisms of ageing and cognitive decline. Nature..

[2] Lv X., Li W., Ma Y., Chen H., Zeng Y., Yu X. (2019 Dec 15). Cognitive decline and mortality among community-dwelling Chinese older people. BMC Med..

[3] Yu B., Steptoe A., Chen Y., Jia X. (2021 Oct 27). Social isolation, rather than loneliness, is associated with cognitive decline in older adults: the China Health and Retirement Longitudinal Study. Psychol Med..

[4] Kwan R.Y.C., Kwan C.W., Kor P.P.K., Chi I. (2022 Mar 16). Cognitive decline, sensory impairment, and the use of audio-visual aids by long-term care facility residents. BMC Geriatr..

[5] World Health Organization (2023). https://www.who.int/news-room/fact-sheets/detail/dementia.

[6] Wrigglesworth J., Ward P., Harding I.H., Nilaweera D., Wu Z., Woods R.L. (2021 Dec 12). Factors associated with brain ageing - a systematic review. BMC Neurol..

[7] Elliott M.L., Belsky D.W., Knodt A.R., Ireland D., Melzer T.R., Poulton R. (2021 Aug 10). Brain-age in midlife is associated with accelerated biological aging and cognitive decline in a longitudinal birth cohort. Mol Psychiatry..

[8] Livingston G., Huntley J., Sommerlad A., Ames D., Ballard C., Banerjee S. (2020 Aug). Dementia prevention, intervention, and care: 2020 report of the Lancet Commission. The Lancet.

[9] World Health Organization (2019).

[10] Dalile B., Kim C., Challinor A., Geurts L., Gibney E.R., Galdos M.V. (2022 Sep). The EAT–Lancet reference diet and cognitive function across the life course. Lancet Planet Health..

[11] Morris M.C., Tangney C.C., Wang Y., Sacks F.M., Barnes L.L., Bennett D.A. (2015 Sep 15). MIND diet slows cognitive decline with aging. Alzheimer’s & Dementia..

[12] van den Brink A.C., Brouwer-Brolsma E.M., Berendsen A.A.M., van de Rest O. (2019 Nov). The Mediterranean, Dietary Approaches to Stop Hypertension (DASH), and Mediterranean-DASH Intervention for Neurodegenerative Delay (MIND) Diets Are Associated with Less Cognitive Decline and a Lower Risk of Alzheimer’s Disease—A Review. Adv Nutr..

[13] Kwan R.Y.C., Law Q.P.S., Tsang J.T.Y., Lam S.H., Wang K.T., Sin O.S.K. (2024 Dec). The Effect of the Mediterranean Diet–Integrated Gamified Home-Based Cognitive-Nutritional (GAHOCON) Training Programme for Older People With Cognitive Frailty: Pilot Randomized Controlled Trial. JMIR Rehabil Assist Technol..

[14] Kwan R.Y.C., Cheung D.S.K., Lo S.K.L., Ho L.Y.W., Katigbak C., Chao Y.Y. (2019 May). Frailty and its association with the Mediterranean diet, life-space, and social participation in community-dwelling older people. Geriatr Nurs (Minneap)..

[15] Ho L.Y.W., Cheung D.S.K., Kwan R.Y.C., Wong A.S.W., Lai C.K.Y. (2021 Mar). Factors associated with frailty transition at different follow-up intervals: a scoping review. Geriatr Nurs (Minneap)..

[16] Liu X., Morris M.C., Dhana K., Ventrelle J., Johnson K., Bishop L. (2021 Mar). Mediterranean-DASH Intervention for Neurodegenerative Delay (MIND) study: Rationale, design and baseline characteristics of a randomized control trial of the MIND diet on cognitive decline. Contemp Clin Trials..

[17] Morris M.C., Tangney C.C., Wang Y., Sacks F.M., Bennett D.A., Aggarwal N.T. (2015 Sep 11). MIND diet associated with reduced incidence of Alzheimer’s disease. Alzheimers Dement..

[18] van Soest A.P., Beers S., van de Rest O., de Groot L.C. (2024 Mar). The Mediterranean-Dietary Approaches to Stop Hypertension Intervention for Neurodegenerative Delay (MIND) Diet for the Aging Brain: A Systematic Review. Adv Nutr..

[19] Berendsen A.M., Kang J.H., Feskens E.J.M., de Groot C.P.G.M., Grodstein F. (2018 Feb). Association of long-term adherence to the mind diet with cognitive function and cognitive decline in American women. J Nutr Health Aging..

[20] Melo van Lent D., O’Donnell A., Beiser A.S., Vasan R.S., DeCarli C.S., Scarmeas N. (2021 Jul 21). Mind diet adherence and cognitive performance in the framingham heart study. J Alzheimers Dis..

[21] de Crom T.O.E., Mooldijk S.S., Ikram M.K., Ikram M.A., Voortman T. (2022 Dec 12). MIND diet and the risk of dementia: a population-based study. Alzheimers Res Ther..

[22] Boumenna T., Scott T.M., Lee J.S., Zhang X., Kriebel D., Tucker K.L. (2022 Mar 3). MIND Diet and Cognitive Function in Puerto Rican Older Adults. J Gerontol Series A..

[23] Hosking D.E., Eramudugolla R., Cherbuin N., Anstey K.J. (2019 Apr 27). MIND not Mediterranean diet related to 12‐year incidence of cognitive impairment in an Australian longitudinal cohort study. Alzheimers Dement..

[24] Chen H., Dhana K., Huang Y., Huang L., Tao Y., Liu X. (2023 Jun 1). Association of the Mediterranean Dietary Approaches to Stop Hypertension Intervention for Neurodegenerative Delay (MIND) Diet With the Risk of Dementia. JAMA Psychiatry..

[25] Arjmand G., Abbas-Zadeh M., Eftekhari M.H. (2022 Feb 21). Effect of MIND diet intervention on cognitive performance and brain structure in healthy obese women: a randomized controlled trial. Sci Rep..

[26] Barnes L.L., Dhana K., Liu X., Carey V.J., Ventrelle J., Johnson K. (2023 Aug 17). Trial of the MIND Diet for Prevention of Cognitive Decline in Older Persons. N Engl J Med..

[27] Kheirouri S., Alizadeh M. (2022 Oct 17). MIND diet and cognitive performance in older adults: a systematic review. Crit Rev Food Sci Nutr..

[28] Devranis P., Vassilopoulou E., Tsironis V., Sotiriadis P.M., Chourdakis M., Aivaliotis M. (2023 Jan 6). Mediterranean diet, ketogenic diet or MIND diet for aging populations with cognitive decline: a systematic review. Life..

[29] Liu Y.H., Gao X., Na M., Kris-Etherton P.M., Mitchell D.C., Jensen G.L. (2020 Oct 27). Dietary pattern, diet quality, and dementia: a systematic review and meta-analysis of prospective cohort studies. J Alzheimers Dis.

[30] Page M.J., McKenzie J.E., Bossuyt P.M., Boutron I., Hoffmann T.C., Mulrow C.D. (2021 Apr). The PRISMA 2020 statement: an updated guideline for reporting systematic reviews. Int J Surg.

[31] Campbell M., McKenzie J.E., Sowden A., Katikireddi S.V., Brennan S.E., Ellis S. (2020 Jan 16). Synthesis without meta-analysis (SWiM) in systematic reviews: reporting guideline. BMJ..

[32] Ouzzani M., Hammady H., Fedorowicz Z., Elmagarmid A. (2016 Dec 5). Rayyan—a web and mobile app for systematic reviews. Syst Rev..

[33] Joanna Briggs Institute. Critical Appraisal Tools. https://jbi.global/critical-appraisal-tools.

[34] Moola S., Munn Z., Tufunaru C., Aromataris E., Sears K., Sfetc R., Aromataris E., Munn Z. (2020). JBI Manual for Evidence Synthesis.

[35] Barker T.H., Stone J.C., Sears K., Klugar M., Tufanaru C., Leonardi-Bee J. (2023 Feb 3). The revised JBI critical appraisal tool for the assessment of risk of bias for randomized controlled trials. JBI Evid Synth..

[36] Shorey S., Ng E.D. (2021 Mar). The use of virtual reality simulation among nursing students and registered nurses: a systematic review. Nurse Educ Today..

[37] Shakersain B., Rizzuto D., Larsson S., Faxén-Irving G., Fratiglioni L., Xu W.L. (2018 Feb 17). The nordic prudent diet reduces risk of cognitive decline in the Swedish older adults: a population-based cohort study. Nutrients.

[38] Adjibade M., Assmann K.E., Julia C., Galan P., Hercberg S., Kesse-Guyot E. (2019 Apr 31). Prospective association between adherence to the MIND diet and subjective memory complaints in the French NutriNet-Santé cohort. J Neurol..

[39] Cherian L., Wang Y., Fakuda K., Leurgans S., Aggarwal N., Morris M. (2019). Mediterranean-DASH Intervention For Neurodegenerative Delay (MIND) Diet Slows Cognitive Decline After Stroke. J Prev Alzheimers Dis..

[40] Mueller K.D., Norton D., Koscik R.L., Morris M.C., Jonaitis E.M., Clark L.R. (2020 Apr 23). Self-reported health behaviors and longitudinal cognitive performance in late middle age: Results from the Wisconsin Registry for Alzheimer’s Prevention. PLoS One..

[41] Munoz-Garcia M.I., Toledo E., Razquin C., Dominguez L.J., Maragarone D., Martinez-Gonzalez J. (2020). “A priori” dietary patterns and cognitive function in the SUN project. Neuroepidemiology..

[42] Dhana K., James B.D., Agarwal P., Aggarwal N.T., Cherian L.J., Leurgans S.E. (2021 Sep 14). MIND diet, common brain pathologies, and cognition in community-dwelling older adults. J Alzheimers Dis..

[43] Nishi S.K., Babio N., Gómez-Martínez C., Martínez-González M.Á, Ros E., Corella D. (2021 Dec 13). Mediterranean, DASH, and MIND dietary patterns and cognitive function: the 2-year longitudinal changes in an older Spanish cohort. Front Aging Neurosci..

[44] Lotan R., Ravona-Springer R., Shakked J., Lin H.M., Ouyang Y., Shahar D.R. (2022 Aug). Greater intake of the MEDI diet is associated with better cognitive trajectory in older adults with type 2 diabetes. Diabetes Res Clin Pract.

[45] Thomas A., Lefèvre-Arbogast S., Féart C., Foubert-Samier A., Helmer C., Catheline G. (2022). Association of a MIND diet with brain structure and dementia in a French population. J Prev Alzheimers Dis..

[46] Vu T., Beck T., Bennett D., Schneider J., Hayden K., Shadyab A. (2022 Jul 3). Adherence to MIND diet, genetic susceptibility, and incident dementia in three US cohorts. Nutrients..

[47] Cornelis M., Agarwal P., Holland T., van Dam R. (2023 Dec 21). MIND dietary pattern and its association with cognition and incident dementia in the UK biobank. Nutrients..

[48] Dong R., Denier-Fields D.N., Van Hulle C.A., Kollmorgen G., Suridjan I., Wild N. (2023 May 25). Identification of plasma metabolites associated with modifiable risk factors and endophenotypes reflecting Alzheimer’s disease pathology. Eur J Epidemiol..

[49] Huang L., Tao Y., Chen H., Chen X., Shen J., Zhao C. (2023 Jul). Mediterranean-Dietary Approaches to Stop Hypertension Intervention for Neurodegenerative Delay (MIND) Diet and Cognitive Function and its Decline: A Prospective Study and Meta-analysis of Cohort Studies. Am J Clin Nutr..

[50] Wagner M., Agarwal P., Leurgans S.E., Bennett D.A., Schneider J.A., Capuano A.W. (2023 Aug 28). The association of MIND diet with cognitive resilience to neuropathologies. Alzheimers Dement..

[51] Zhang J., Cao X., Li X., Li X., Hao M., Xia Y. (2023 Jul). Associations of Midlife Dietary Patterns with Incident Dementia and Brain Structure: Findings from the UK Biobank Study. Am J Clin Nutr..

[52] McEvoy C.T., Guyer H., Langa K.M., Yaffe K. (2017 Aug 25). Neuroprotective Diets Are Associated with Better Cognitive Function: The Health and Retirement Study. J Am Geriatr Soc..

[53] Calil S.R.B., Brucki S.M.D., Nitrini R., Yassuda M.S. (2018 Dec). Adherence to the Mediterranean and MIND diets is associated with better cognition in healthy seniors but not in MCI or AD. Clin Nutr ESPEN..

[54] Wesselman L.M.P., van Lent D.M., Schröder A., van de Rest O., Peters O., Menne F. (2021 Mar 29). Dietary patterns are related to cognitive functioning in elderly enriched with individuals at increased risk for Alzheimer’s disease. Eur J Nutr..

[55] Ahn S., Lingerfelt C.N., Lee C.E., Lee J.A., Raynor H.A., Anderson J.G. (2022 Jul). Association of adherence to high-intensity physical activity and the Mediterranean-dietary approaches to stop hypertension intervention for neurodegenerative delay diet with cognition: a cross-sectional study. Int J Nurs Stud..

[56] Ferreira N.V., Lotufo P.A., Marchioni D.M.L., Barreto S.M., Viana M.C., Caramelli P. (2022 Apr). Association between adherence to the MIND diet and cognitive performance is affected by income. Alzheimer Dis Assoc Disord..

[57] Gauci S., Young L.M., Arnoldy L., Scholey A., White D.J., Lassemillante A.C. (2022 Apr 28). RETRACTED: the association between diet and cardio-metabolic risk on cognitive performance: a cross-sectional study of middle-aged Australian adults. Front Nutr..

[58] Huang X., Aihemaitijiang S., Ye C., Halimulati M., Wang R., Zhang Z. (2022 Aug). Development of the cMIND diet and its association with cognitive impairment in older Chinese people. J Nutr Health Aging..

[59] Lawrie S., Coe S., Mansoubi M., Welch J., Razzaque J., Hu M.T. (2023 May 19). Dietary patterns and nonmotor symptoms in Parkinson’s disease: a cross-sectional analysis. J Am Nutr Assoc..

[60] Vassilopoulou E., Koumbi L., Karastogiannidou C., Sotiriadis P.M., Felicia P.C., Tsolaki M. (2022 Sep 7). Adjustment of the MIND diet tool for discriminating Greek patients with dementia: a confirmatory factor analysis. Front Neurol..

[61] Yeung S.S.Y., Sin D., Yu R., Leung J., Woo J. (2022 Feb). Dietary patterns and intrinsic capacity in community-dwelling older adults: a cross-sectional study. J Nutr Health Aging..

[62] Escher C E., Asken B.M., VandeBunte A., Fonseca C., You M. (2023 Feb 17). Roles of physical activity and diet in cognitive aging: is more better?. Clin Neuropsychol..

[63] Zare S., Eftekhari M.H., Arjmand G., Zare M. (2023). Adherence to Mediterranean-Dash Intervention for Neurodegenerative Delay (MIND) Dietary Pattern in Elderly with Type 2 Diabetes and the Correlation with Cognitive Functions and Metabolic Profile. Int J Nutr Sci..

[64] Derdiger S., Friedeborn S., Tangney C.C. (2024 Feb). Cognitive performance in relation to MIND and MEPA III dietary pattern accordance of NHANES participants. J Human Nutr Dietetics..

[65] Filippini T., Adani G., Malavolti M., Garuti C., Cilloni S., Vinceti G. (2020 Nov 29). Dietary habits and risk of early-onset dementia in an Italian case-control study. Nutrients..

[66] Chu C.Q., lei Yu L., Yuan Qi G., Mi Y.S., Wu W.Q., Kun Lee Y. (2022). Can dietary patterns prevent cognitive impairment and reduce Alzheimer’s disease risk: exploring the underlying mechanisms of effects. Neurosci Biobehav Rev.

[67] Morris M.C., Evans D.A., Tangney C.C., Bienias J.L., Wilson R.S. (2006 Oct 24). Associations of vegetable and fruit consumption with age-related cognitive change. Neurology..

[68] Devore E.E., Kang J.H., Breteler M.M.B., Grodstein F. (2012 Jul 26). Dietary intakes of berries and flavonoids in relation to cognitive decline. Ann Neurol..

[69] Cannon-Albright L.A., Foster N.L., Schliep K., Farnham J.M., Teerlink C.C., Kaddas H. (2019 Apr 9). Relative risk for Alzheimer disease based on complete family history. Neurology..

[70] Morris M.C. (2016 Mar 26). Nutrition and risk of dementia: overview and methodological issues. Ann N Y Acad Sci..

[71] Morris M.C., Tangney C.C. (2014 Sep). Dietary fat composition and dementia risk. Neurobiol Aging.

[72] Zachariou V., Bauer C.E., Seago E.R., Panayiotou G., Hall E.D., Butterfield D.A. (2021 Oct). Healthy dietary intake moderates the effects of age on brain iron concentration and working memory performance. Neurobiol Aging..

[73] Talegawkar S.A., Jin Y., Simonsick E.M., Tucker K.L., Ferrucci L., Tanaka T. (2022 Mar). The Mediterranean-DASH Intervention for Neurodegenerative Delay (MIND) diet is associated with physical function and grip strength in older men and women. Am J Clin Nutr..

[74] Lim G.P., Calon F., Morihara T., Yang F., Teter B., Ubeda O. (2005 Mar 23). A diet enriched with the omega-3 fatty acid docosahexaenoic acid reduces amyloid burden in an aged Alzheimer mouse model. J Neurosci..

[75] McHugh J.E., Lawlor B.A. (2016 Apr 2). Executive functioning independently predicts self-rated health and improvement in self-rated health over time among community-dwelling older adults. Aging Ment Health..

[76] Higgins J.P.T., Thomas J., Chandler J., Cumpston M., Li T., Page M.J. (2019).

[77] Cumpston M.S., McKenzie J.E., Welch V.A., Brennan S.E. (2022 Dec 1). Strengthening Systematic Reviews in public health: guidance in the Cochrane Handbook for Systematic Reviews of Interventions, 2nd ed. J Public Health (Bangkok).

[78] Moss J., De Bin R. (2023 Mar 27). Modelling publication bias and p ‐hacking. Biometrics..

