# Supplementary material for: The association between the MIND diet and cognitive health in middle-aged and older adults: A systematic review
Source: J Nutr Health Aging. 2025 Jul 10;29(9):100630. doi: 10.1016/j.jnha.2025.100630 (PMC12695522; doi:10.1016/j.jnha.2025.100630)
Supplement: Supplementary file 1 [file mmc1.docx]

| **Appendix I. Search strategy using CINAHL Complete, PubMed, Cochrane Library, Web of Science and EMBASE.** | | | |
| --- | --- | --- | --- |
| Database |  | Search terms | Number of studies searched |
| CINAHL Complete (EBSCOhost Research Databases) | S1 | MIND diet OR Mediterranean-DASH Diet Intervention for Neurodegenerative Delay OR Neurodegenerative Delay diet |  |
|  | S2 | MH "MIND diet+" OR MH "Mediterranean-DASH Diet Intervention for Neurodegenerative Delay+" OR Neurodegenerative Delay diet |  |
|  | S3 | TX "MIND diet" OR TX "Mediterranean-DASH Diet Intervention for Neurodegenerative Delay" OR TX "Neurodegenerative Delay diet" |  |
|  | S4 | ( MH "middle age+" OR MH "middle-age+" ) OR ( MH "middle aged+" OR MH "middle-aged+" ) OR ( "adult*" OR "older adult*" OR "older person*" OR "older people" ) |  |
|  | S5 | MH "aged+" OR ( "old aged" OR "old-aged" ) OR ( "elderly" or "elderly people" or "senior" ) |  |
|  | S6 | S4 OR S5 |  |
|  | S7 | MH "dementia+" OR ( MH "Alzheimer's Disease+" OR MH "Alzheimer Disease+" OR MH "Alzheimer+" OR "AD" ) OR ( "Mild cognitive impairment" OR "MCI" ) OR ( MH "Cognitive Dysfunction+" OR "Cognitive Dysfunction*" ) OR ( "cognitive impairment*" OR "cognitive disorder*" ) |  |
|  | S8 | "learning" OR "cognition" OR "cognitive" OR "memory" OR "executive function" OR "priming" |  |
|  | S9 | S7 OR S8 |  |
|  | S10 | S1 OR S2 OR S3 |  |
|  | S11 | S6 AND S9 AND S10 | 258 |
| PubMed | 1 | MIND diet |  |
|  | 2 | MIND diet[MeSH Terms] |  |
|  | 3 | Mediterranean-DASH Diet Intervention for Neurodegenerative Delay[MeSH Terms] |  |
|  | 4 | Mediterranean-DASH Diet Intervention for Neurodegenerative Delay |  |
|  | 5 | Neurodegenerative Delay diet[MeSH Terms] |  |
|  | 6 | #2 OR #4 OR #5 |  |
|  | 7 | (((((((middle age[MeSH Terms]) OR (middle-age[MeSH Terms])) OR (middle aged[MeSH Terms])) OR (middle-aged[MeSH Terms])) OR (adult*[MeSH Terms])) OR (older adult*[MeSH Terms])) OR (older person*[MeSH Terms])) OR (older people[MeSH Terms]) |  |
|  | 8 | (((((aged[MeSH Terms]) OR (old aged[MeSH Terms])) OR (old-aged[MeSH Terms])) OR (elderly[MeSH Terms])) OR (elderly people[MeSH Terms])) OR (senior[MeSH Terms]) |  |
|  | 9 | #7 OR #8 |  |
|  | 10 | (((((((((dementia[MeSH Terms]) OR (Alzheimer's Disease[MeSH Terms])) OR (Alzheimer Disease[MeSH Terms])) OR (Alzheimer[MeSH Terms])) OR (Mild cognitive impairment[MeSH Terms])) OR (MCI[MeSH Terms])) ) OR (Cognitive Dysfunction*[MeSH Terms])) OR (cognitive impairment*[MeSH Terms])) OR (cognitive disorder*"[MeSH Terms]) |  |
|  | 11 | learning OR cogniti* OR memory OR executive function OR priming |  |
|  | 12 | #10 OR #11 |  |
|  | 13 | #6 AND #9 AND #12 | 96 |
| Cochrane Library | 1 | (MIND diet) OR (Mediterranean-DASH Diet Intervention for Neurodegenerative Delay) OR (Neurodegenerative Delay diet) (Word variations have been searched) with Cochrane Library publication date Between Jan 2015 and Apr 2023 |  |
|  | 2 | MeSH descriptor: [Middle Aged] explode all trees |  |
|  | 3 | MeSH descriptor: [Frail Elderly] explode all trees |  |
|  | 4 | MeSH descriptor: [Aged] explode all trees |  |
|  | 5 | (middle age):ti,ab,kw OR (middle-age):ti,ab,kw OR (middle aged):ti,ab,kw OR (middle-aged):ti,ab,kw (Word variations have been searched) with Cochrane Library publication date Between Jan 2015 and Apr 2023 |  |
|  | 6 | (adult*):ti,ab,kw OR (older adult*):ti,ab,kw OR (older person*):ti,ab,kw OR (older people):ti,ab,kw (Word variations have been searched) with Cochrane Library publication date Between Jan 2015 and Apr 2023 |  |
|  | 7 | (aged):ti,ab,kw OR (old aged):ti,ab,kw OR (old-aged):ti,ab,kw OR (elderly*):ti,ab,kw OR (senior):ti,ab,kw (Word variations have been searched) with Cochrane Library publication date Between Jan 2015 and Apr 2023 |  |
|  | 8 | #2 OR #3 OR #4 OR #5 OR #6 OR #7 with Cochrane Library publication date Between Jan 2015 and Apr 2023 (Word variations have been searched) |  |
|  | 9 | MeSH descriptor: [Dementia] explode all trees |  |
|  | 10 | MeSH descriptor: [Alzheimer Disease] explode all trees |  |
|  | 11 | MeSH descriptor: [Cognitive Dysfunction] explode all trees |  |
|  | 12 | MeSH descriptor: [Cognition Disorders] explode all trees |  |
|  | 13 | (dementia):ti,ab,kw OR (Alzheimer's Disease):ti,ab,kw OR (Alzheimer Disease):ti,ab,kw OR (Alzheimer):ti,ab,kw (Word variations have been searched) with Cochrane Library publication date Between Jan 2015 and Apr 2023 |  |
|  | 14 | (Mild cognitive impairment):ti,ab,kw OR (MCI):ti,ab,kw OR (cognitive Dysfunction*):ti,ab,kw OR (cognitive impairment*):ti,ab,kw OR (cognitive disorder*):ti,ab,kw (Word variations have been searched) with Cochrane Library publication date Between Jan 2015 and Apr 2023 |  |
|  | 15 | (learning):ti,ab,kw OR (cogniti*):ti,ab,kw OR (memory):ti,ab,kw OR (executive function):ti,ab,kw OR (priming):ti,ab,kw (Word variations have been searched) with Cochrane Library publication date Between Jan 2015 and Apr 2023 |  |
|  | 16 | #9 OR #10 OR #11 OR #12 OR #13 OR #14 OR #15 with Cochrane Library publication date Between Jan 2015 and Apr 2023 (Word variations have been searched) |  |
|  | 17 | #1 AND #8 AND #16 with Cochrane Library publication date Between Jan 2015 and Apr 2023 (Word variations have been searched) | 182 |
| Web of Science Core Collection | 1 | ALL=(MIND diet) OR ALL=(Mediterranean-DASH Diet Intervention for Neurodegenerative Delay) OR ALL=(Neurodegenerative Delay diet) |  |
|  | 2 | (((((((ALL=(middle age)) OR ALL=(middle-age)) OR ALL=(middle aged)) OR ALL=(middle-aged)) OR ALL=(adult*)) OR ALL=(older adult*)) OR ALL=(older person*)) OR ALL=(older people) |  |
|  | 3 | (((((ALL=(aged)) OR ALL=(old aged)) OR ALL=(old-aged)) OR ALL=(elderly)) OR ALL=(elderly people)) OR ALL=(senior) |  |
|  | 4 | #2 OR #3 |  |
|  | 5 | ((((((((ALL=(dementia)) OR ALL=(Alzheimer's Disease)) OR ALL=(Alzheimer Disease)) OR ALL=(Alzheimer)) OR ALL=(Mild cognitive impairment)) OR ALL=(MCI)) OR ALL=(Cognitive Dysfunction*)) OR ALL=(Cognitive impairment*)) OR ALL=(Cognitive disorder*) |  |
|  | 6 | ((((ALL=(learning)) OR ALL=(cogniti*)) OR ALL=(memory)) OR ALL=(executive function)) OR ALL=(priming) |  |
|  | 7 | #5 OR #6 |  |
|  | 8 | #1 AND #4 AND #7 | 278 |
| EMBASE | 1 | MIND diet.mp. or exp MIND diet/ |  |
|  | 2 | Mediterranean-DASH Diet Intervention for Neurodegenerative Delay.mp. |  |
|  | 3 | Neurodegenerative Delay diet.mp. |  |
|  | 4 | 1 or 2 or 3 |  |
|  | 5 | exp middle aged/ or exp aging/ or exp adult/ or middle$age*.mp. or exp aged/ |  |
|  | 6 | older person*.mp. |  |
|  | 7 | older people.mp. |  |
|  | 8 | older adult*.mp. |  |
|  | 9 | old$age*.mp. |  |
|  | 10 | elderly.mp. |  |
|  | 11 | senior.mp. |  |
|  | 12 | elderly people.mp. |  |
|  | 13 | 5 or 6 or 7 or 8 or 9 or 10 or 11 or 12 |  |
|  | 14 | Alzheimer's Disease.mp. or exp Alzheimer disease/ |  |
|  | 15 | Alzheimer.mp. |  |
|  | 16 | exp dementia/ or dementia.mp. |  |
|  | 17 | mild cognitive impairment.mp. or exp mild cognitive impairment/ |  |
|  | 18 | cognitive dysfunction.mp. or exp cognitive defect/ |  |
|  | 19 | 14 or 15 or 16 or 17 or 18 |  |
|  | 20 | learning.mp. or exp learning/ |  |
|  | 21 | exp cognition/ or cogniti*.mp. |  |
|  | 22 | memory.mp. or exp memory/ |  |
|  | 23 | executive function.mp. or exp executive function/ |  |
|  | 24 | priming.mp. |  |
|  | 25 | 20 or 21 or 22 or 23 or 24 |  |
|  | 26 | 4 and 13 and 19 and 25 |  |
|  | 27 | limit 26 to yr="2015 - 2023" | 84 |

**Appendix II. The JBI Critical Appraisal Checklists**^34,35^

**JBI Critical Appraisal Checklist for cohort studies**

|  | Yes | No | Unclear | Not applicable |
| --- | --- | --- | --- | --- |
| 1. Were the two groups similar and recruited from the same population? | □ | □ | □ | □ |
| 1. Were the exposures measured similarly to assign people to both exposed and unexposed groups? | □ | □ | □ | □ |
| 1. Was the exposure measured in a valid and reliable way? | □ | □ | □ | □ |
| 1. Were confounding factors identified? | □ | □ | □ | □ |
| 1. Were strategies to deal with confounding factors stated? | □ | □ | □ | □ |
| 1. Were the groups/participants free of the outcome at the start of the study (or at the moment of exposure)? | □ | □ | □ | □ |
| 1. Were the outcomes measured in a valid and reliable way? | □ | □ | □ | □ |
| 1. Was the follow up time reported and sufficient to be long enough for outcomes to occur? | □ | □ | □ | □ |
| 1. Was follow up complete, and if not, were the reasons to loss to follow up described and explored? | □ | □ | □ | □ |
| 1. Were strategies to address incomplete follow up utilized? | □ | □ | □ | □ |
| 1. Was appropriate statistical analysis used? | □ | □ | □ | □ |

**Appendix II. The JBI Critical Appraisal Checklists**^34,35^ **(cont’d)**

**JBI Critical Appraisal Checklist for
analytical cross sectional studies**

Reviewer ______________________________________ Date_______________________________

Author_______________________________________ Year_________ Record Number_________

|  | Yes | No | Unclear | Not applicable |
| --- | --- | --- | --- | --- |
| 1. Were the criteria for inclusion in the sample clearly defined? | □ | □ | □ | □ |
| 1. Were the study subjects and the setting described in detail? | □ | □ | □ | □ |
| 1. Was the exposure measured in a valid and reliable way? | □ | □ | □ | □ |
| 1. Were objective, standard criteria used for measurement of the condition? | □ | □ | □ | □ |
| 1. Were confounding factors identified? | □ | □ | □ | □ |
| 1. Were strategies to deal with confounding factors stated? | □ | □ | □ | □ |
| 1. Were the outcomes measured in a valid and reliable way? | □ | □ | □ | □ |
| 1. Was appropriate statistical analysis used? | □ | □ | □ | □ |

**Appendix II. The JBI Critical Appraisal Checklists**^34,35^ **(cont’d)**

**JBI Critical Appraisal Checklist for
case control studies**

Reviewer ______________________________________ Date_______________________________

Author_______________________________________ Year_________ Record Number_________

|  | Yes | No | Unclear | Not applicable |
| --- | --- | --- | --- | --- |
| 1. Were the groups comparable other than the presence of disease in cases or the absence of disease in controls? | □ | □ | □ | □ |
| 1. Were cases and controls matched appropriately? | □ | □ | □ | □ |
| 1. Were the same criteria used for identification of cases and controls? | □ | □ | □ | □ |
| 1. Was exposure measured in a standard, valid and reliable way? | □ | □ | □ | □ |
| 1. Was exposure measured in the same way for cases and controls? | □ | □ | □ | □ |
| 1. Were confounding factors identified? | □ | □ | □ | □ |
| 1. Were strategies to deal with confounding factors stated? | □ | □ | □ | □ |
| 1. Were outcomes assessed in a standard, valid and reliable way for cases and controls? | □ | □ | □ | □ |
| 1. Was the exposure period of interest long enough to be meaningful? | □ | □ | □ | □ |
| 1. Was appropriate statistical analysis used? | □ | □ | □ | □ |

**Appendix II. The JBI Critical Appraisal Checklists**^34,35^ **(cont’d)**

**JBI Critical Appraisal Checklist for
randomized Controlled trials**

Reviewer ______________________________________ Date_______________________________

Author_______________________________________ Year_________ Record Number_________

|  | Yes | No | Unclear | NA |
| --- | --- | --- | --- | --- |
| 1. Was true randomization used for assignment of participants to treatment groups? | □ | □ | □ | □ |
| 1. Was allocation to treatment groups concealed? | □ | □ | □ | □ |
| 1. Were treatment groups similar at the baseline? | □ | □ | □ | □ |
| 1. Were participants blind to treatment assignment? | □ | □ | □ | □ |
| 1. Were those delivering treatment blind to treatment assignment? | □ | □ | □ | □ |
| 1. Were outcomes assessors blind to treatment assignment? | □ | □ | □ | □ |
| 1. Were treatment groups treated identically other than the intervention of interest? | □ | □ | □ | □ |
| 1. Was follow up complete and if not, were differences between groups in terms of their follow up adequately described and analyzed? | □ | □ | □ | □ |
| 1. Were participants analyzed in the groups to which they were randomized? | □ | □ | □ | □ |
| 1. Were outcomes measured in the same way for treatment groups? | □ | □ | □ | □ |
| 1. Were outcomes measured in a reliable way? | □ | □ | □ | □ |
| 1. Was appropriate statistical analysis used? | □ | □ | □ | □ |
| 1. Was the trial design appropriate, and any deviations from the standard RCT design (individual randomization, parallel groups) accounted for in the conduct and analysis of the trial? | □ | □ | □ | □ |

**Appendix III.**

Detailed methods used in included studies to calculate global cognitive scores and administer domain-specific cognitive tests (follows the sequence of Table 1 for easy reference).

| **No. in Table 1** | **First author (year), country** | **Outcome (Assessment methods)** | **Detailed methods** |
| --- | --- | --- | --- |
| 2 | Morris (2015), USA | 1. Global cognitive function | Composite scores were generated for each cognitive domain and a global measure covering all 19 tests. Raw scores were standardized using the baseline population's mean and standard deviation, then averaged. |
|  |  | 2. Domain-specific cognitive functions  (episodic memory, working memory, semantic memory, visuospatial ability, and perceptual speed) | **Episodic Memory:** Word List Memory Word List Recall Word List Recognition Immediate recall of the East Boston Story Delayed recall of the East Boston Story Immediate recall of Story A from Logical Memory (Wechsler Memory Scale-Revised) Delayed recall of Story A from Logical Memory (Wechsler Memory Scale-Revised)  **Working Memory:** Digit Span Forward (Wechsler Memory Scale-Revised) Digit Span Backward (Wechsler Memory Scale-Revised) Digit Ordering  **Semantic Memory:** 15-item form of the Boston Naming Test Verbal Fluency 15-item version of the National Adult Reading Test  **Visuospatial Ability:** 15-item form of Judgment of Line Orientation 16-item form of Standard Progressive Matrices  **Perceptual Speed:** Oral version of the Symbol Digit Modalities Test Number Comparison Modified Stroop Neuropsychological Screening Test: Number of color names correctly read minus names incorrectly read Number of colors correctly named minus colors incorrectly named in a 30-second trial |
| 3 | Berendsen (2018), USA | 1. Global cognitive function | The global cognitive score was derived by averaging the z-scores from the cognitive tests. The z-scores for each follow-up were computed using the means and standard deviations from the initial cognitive assessment. |
|  |  | 2. Domain-specific cognitive functions  (TICS; Verbal (Episodic) Memory; Language and Executive Function; Working Memory and Attention) | **TICS (Telephone Interview for Cognitive Status): 0–41 points** Adaptation of the Mini-Mental State Examination  **Verbal (Episodic) Memory:** EBMT (East Boston Memory Test): 0–12 points TICS 10-word list delayed recall: 0–10 points  **Language and Executive Function:** Category Fluency Test: Naming as many animals as possible in one minute  **Working Memory and Attention:** Digit-span Backward Test: 0–12 points Participants repeat an increasingly long series of digits backwards |
| 4 | Shakersain (2018), Sweden | 1. Global cognitive function | Cognitive function was evaluated with the MMSE for overall cognition at the start and during each follow-up. A significant cognitive decline was defined by an MMSE score dropping to 24 or below. |
| 6 | Cherian (2019), USA | 1. Global cognitive function | Standardized scores for each test were calculated using baseline means and standard deviations, then averaged across each cognitive domain and all tests to form a global cognitive score. |
|  |  | 2. Domain-specific cognitive functions (episodic memory; semantic memory; working memory; perceptual orientation; perceptual speed) | **Episodic Memory:** Word List Word List Recall Word List Recognition East Boston Immediate Recall East Boston Delayed Recall Logical Memory I (Immediate) Logical Memory II (Delayed)  **Semantic Memory:** Boston Naming (15 items) Category Fluency Reading Test (10 items)  **Working Memory:** Digits Forward Digits Backward Digit Ordering  **Perceptual Orientation:** Line Orientation Progressive Matrices (16 items)  **Perceptual Speed:** Symbol Digits Modality-Oral Number Comparison Stroop Color Naming Stroop Word Reading |
| 8 | Mueller (2020), USA | 1. Global cognitive function (PACC4 - RAVLT; Logical Memory II subtest from WMS-R; Digit Substitution test of the WAIS-R; MMSE) | **PACC4 Composite Score Components:**  Averaged standardized test scores (z-scores) from: **Rey Auditory Verbal Learning Test (RAVLT):** Total recall learning trials 1–5 **Logical Memory II subtest (WMS-R):** Delayed recall of stories A and B **Digit Substitution test (WAIS-R):** Total scores **Mini-Mental Status Examination (MMSE):** Total scores |
|  |  | 2. Domain-specific cognitive functions (Immediate Recall; Delayed Recall; Executiive functioning) | **Immediate Recall:** RAVLT total from trials 1–5 WMS-R LM Immediate Recall, stories A and B BVMT-R Immediate Recall, trials 1–3  **Delayed Recall:** RAVLT Long-Delay Free Recall WMS-R LM Delayed Recall BVMT-R Delayed Recall  **Executive Functioning:** Trail Making Test Part B total time to completion (z-score multiplied by -1 for consistency) Stroop Neuropsychological Screening Test, color-word interference total items completed in 120 seconds WAIS-R Digit Symbol Coding total items completed in 90 seconds |
| 9 | Munoz-Garcia (2020), Spain | 1. Domain-specific cognitive functions (STICS-m, telephone adaptation of the MMSE) | **STICS-m**, a telephone-based version of the Mini-Mental State Examination. The STICS-m assesses four cognitive domains: **orientation, memory, attention/calculation, and language,** with a maximum score of 41. |
| 10 | Dhana (2021), USA | 1. Global cognitive function (Episodic memory; semantic memory; working memory; perceptual speed, and visuospatial ability) | Nineteen neuropsychological tests were utilized to develop a composite measure of global cognitive function, covering five specific domains: **episodic memory, semantic memory, working memory, perceptual speed, and visuospatial ability**. Raw scores from each test were standardized and averaged into z-scores, using the mean and standard deviation from the baseline evaluation across the study. |
| 11 | Melo van Lent (2021), USA | 1. Global cognitive function | Task scores were converted into z-scores and summed by their assigned weightings to examine a composite measure of general cognitive function. This score integrates weighted contributions from the **Trail-Making Test Part B, Hooper Visual Organization Test, Logical Memory, Visual Reproductions, Paired Associate Learning, and Similarities**. |
|  |  | 2. Domain-specific cognitive functions (Visual Memory; Meaningful/Verbal Memory; Verbal Comprehension/Reasoning; Processing Speed; Processing Speed and Executive Function; Closure/Visual Integration and Mental Rotation) | **Visual Memory:** Visual Reproductions Delayed Recall (Wechsler Memory Scale)  **Meaningful/Verbal Memory:** Logical Memory Delayed Recall (Wechsler Memory Scale)  **Verbal Comprehension/Reasoning:** Similarities (Wechsler Adult Intelligence Scale)  **Processing Speed:** Trail Making Test A  **Processing Speed and Executive Function:** Trail Making Test B minus A  **Closure/Visual Integration and Mental Rotation:** Hooper Visual Organization Test |
| 12 | Nishi (2021), Spain | 1. Global cognitive function | Global cognitive function (GCF) was determined as a composite score of all eight assessments. |
|  |  | 2. Domain-specific cognitive functions (MMSE; CDT; VFT-a and VFT-p; TMT A&B; DST-b and DST-f) | **Global Cognitive Screening:** Mini-Mental State Examination (MMSE)  **Visuospatial and Visuo-Constructive Capacity:** Clock-Drawing Test (CDT)  **Verbal Ability and Executive Function:** Semantic Verbal Fluency Task (VFT-a) Phonological Verbal Fluency Task (VFT-p)  **Executive Function, Attention, and Processing Speed:** Trail Making Test Part A (attention and processing speed) Trail Making Test Part B (cognitive flexibility)  **Attention, Short-Term Memory, and Working Memory:** Forward Digit Span Test (DST-f) (attention and short-term memory) Backward Digit Span Test (DST-b) (working memory) |
| 13 | Boumenna (2022), USA | 1. Global cognitive function (MMSE; 16-word list learning task; digit span forward and backward; stroop test; clock drawing and figure copying; verbal fluency) | **Overall Cognitive Function:** Mini-Mental State Examination (MMSE)  **Verbal Memory:** 16-word list learning task  **Attention and Working Memory:** Digit Span Forward and Backward  **Processing Speed, Cognitive Flexibility, and Response Inhibition:** Stroop Test  **Visual-Spatial Organization:** Clock Drawing Figure Copying  **Verbal Ability and Executive Function:** Verbal Fluency  Individual test scores were transformed to Z-scores and a global cognitive function composite score (GCS). |
| 15 | Lotan (2022), Israel | 1. Global cognitive function | Each cognitive test score was converted to a z-score using the baseline mean and standard deviation. These z-scores were averaged per domain, then normalized again to create domain-specific composite scores. The global cognition z-score was derived by normalizing the averaged domain z-scores. |
|  |  | 2. Domain-specific cognitive functions (Episodic memory; attention/working memory; language/semantic categorization; executive function) | **Overall Cognitive Function:** Mini-Mental State Examination (MMSE)  **Episodic Memory:** Immediate recall, delayed recall, and recognition (Word List from ADAS-COG)  **Attention/Working Memory:** Shape Cancellation Digit Span Forward and Backward  **Language/Semantic Categorization:** Similarities Animal Fluency 15-item Boston Naming Test  **Executive Function:** Trail Making Test A and B Praxis (from ADAS-COG) Digit Symbol Substitution Test |
| 17 | Vu (2022), USA | 1. Global cognitive function - CHAP & MAP: a battery of cognitive tests; - WHIMS: 3MSE | CHAP & MAP: A set of cognitive tests was administered by trained research staff or nurses during in-home assessments (CHAP) or by psychologists or nurses during clinical visits (MAP). A global cognitive measure was calculated at each clinical visit by converting raw test scores to standardized scores using the baseline population's mean and standard deviation, and then averaging these standardized scores.  WHIMS: Participants were screened annually using the Modified Mini-Mental State Examination (3MSE), which provides a global cognitive score from 0 to 100. Those scoring 80 or below (for participants with 8 or fewer years of education) or 88 or below (for those with 9 or more years of education) underwent additional in-person cognitive testing. |
| 19 | Cornelis (2023), UK | 2. Domain-specific cognitive functions (PM; FI; Pairs matching; RT; SDS; Trail A/Trail B) | **Touchscreen Tests at Assessment Centers (2006–2010):** Prospective Memory (PM) Fluid Intelligence (FI, verbal-numerical reasoning) Pairs Matching (Pairs, visual memory) Reaction Time (RT)  **Online Tests at Home (starting in 2014):** Symbol Digit Substitution (SDS, complex processing speed) Trail Making A and B (visual search, scanning, processing speed, mental flexibility, and executive functions) |
| 20 | Dong (2023), USA | 1. Global cognitive function (PACC; RAVLT; WMS-RLM; BVMT-R; TMT B; Stroop Neuropsychological Screening Test; WAIS-R) | **PACC4 Composite Score Components:** Averaged standardized test scores (z-scores) from: Rey Auditory Verbal Learning Test (RAVLT) trials 1–5; Logical Memory II subtest (delayed recall of stories A and B) from WMS-R; Digit Substitution test from WAIS-R; Mini-Mental Status Examination (MMSE)  **Immediate Recall:** RAVLT total from trials 1–5 WMS-R LM Immediate Recall, stories A and B BVMT-R Immediate Recall, trials 1–3  **Delayed Recall:** RAVLT Long-Delay Free Recall WMS-R LM Delayed Recall BVMT-R Delayed Recall  **Executive Functioning:** Trail Making Test Part B total time to completion (z-score multiplied by -1 for consistency) Stroop Neuropsychological Screening Test, color-word interference total items completed in 120 seconds WAIS-R Digit Symbol Coding total items completed in 90 seconds |
| 21 | Huang (2023), China | 1. Global cognitive function (TICSm) | **Telephone Interview for Cognitive Status-Modified (TICSm):** **Memory:** Immediate and delayed recall of a 10-word list (10 points each) **Attention:** Counting backward from 20 to 1 (2 points) **Calculation Abilities:** Serial 7 subtraction (5 points)  The global cognitive score was calculated by summing all cognitive test item scores, with a possible range of 0 to 27 points. |
|  |  | 2. Verbal memory | The verbal memory score combined the immediate and delayed recalls of a 10-word list, ranging from 0 to 20 points. |
| 22 | Wagner (2023), USA | 1. Domain-specific cognitive functions (Cognitive resilience - 17 psychometric tests administered annually until death; 9 measures of post mortem neuropathology) | Cognitive Resilience (CR)  **17 psychometric tests** Administered annually until death Raw scores converted to z-scores using baseline mean and SD Z-scores averaged at each assessment Neuropathologies Examined at Autopsy:  **9 measures of neuropathologies examined at autopsy:** Hippocampal sclerosis Neocortical Lewy bodies TDP-43 protein Chronic macroscopic and microinfarcts Arteriolosclerosis Atherosclerosis Cerebral amyloid angiopathy |
| 24 | McEvoy (2017), USA | 1. Global cognition function (Immediate and delayed recall; backward counting; serial seven subtraction) | Global Cognition Score Components: **Immediate and delayed recall of 10-word list (0–20 points)** **Backward counting (0–2 points)** **Serial seven subtraction (0–5 points)** Score Range: 0 to 27   Clinically significant poor cognitive performance was defined as scoring one or more standard deviations below the mean global cognition score. |
| 25 | Calil (2018), Brazil | 1.Domain-specific cognitive functions  (MMSE; BCSB; VF; CDT; GDS) | **Mini-Mental State Examination (MMSE)**  **Brief Cognitive Screening Battery (BCSB)** Naming of 10 black and white figures Incidental Memory: Recall named figures Immediate Memory: Study and recall figures after 30 seconds Learning: Repeat procedure and recall Delayed Recall: Recall figures after completing VF and CDT Recognition: Identify 10 target figures among distractors  **Verbal Fluency (VF) - Animal Category  Clock Drawing Test (CDT)  Geriatric Depression Scale (GDS)** |
| 26 | Wesselman (2021), Germany | 1. Domain-specific cognitive functions  (Memory; language; executive function; working memory; visuospatial function) | **Memory:** Alzheimer's Disease Assessment Scale—Cognitive Subscale: word list (trials 1, 2, 3), delayed recall, and recognition Free and Cued Selective Reminding Test (FCSRT): free recall and cue efficiency Wechsler Memory Scale: logical memory 1 + 2 Consortium to Establish a Registry for Alzheimer’s Disease (CERAD): figure savings Symbol-Digit-Modalities Test (SDMT): incidental learning Face Name Test  **Language:** Verbal Fluency: groceries and animals Boston Naming Test (20 items) FCSRT: naming  **Executive Functioning:** Trail Making Test A + B Number Cancellation SDMT Flanker Task  **Working Memory:** Digit Span Forward + Backward FCSRT: interference task (Serial 3s)  **Visuospatial Functioning:** Clock copying + drawing CERAD Figure copying |
| 27 | Ahn (2022), USA | 1. Global cognitive function  (Episodic memory; Working memory; Attention/processing speed) | Global Cognitive Assessment:  **Episodic Memory: Immediate and delayed recall** Recall from a list of 10 words immediately and after 5 minutes (0 to 20 points)  **Working Memory: Serial seven subtraction** Subtract 7 from 100, up to five times (0 to 5 points)  **Attention/Processing Speed: Backward counting** Count backward from 20 quickly (0 to 2 points) |
| 28 | Ferreira (2022), Brazil | 1. Global cognition function | Z-scores for each of the six tasks were created by subtracting the sample mean from the participant's score and dividing by the sample standard deviation. The global cognition composite z-score was then calculated by averaging these task z-scores and standardizing the mean. |
|  |  | 2. Domain-specific cognitive functions  (Memory Domain; Language and Executive Function; Executive Function and Visual-spatial Organization) | **Memory Domain (word list from the Consortium to Establish a Registry for Alzheimer’s Disease - WL-CERAD):** **Word List Learning:** Read 10 unrelated words and recall after three presentations **Word List Delayed Recall:** Recall words 5 minutes after last presentation **Word List Recognition:** Recognize words among 10 distractors Score: Based on words recalled (higher is better)  **Language and Executive Function:** **Semantic Fluency:** List as many animals as possible in one minute **Phonemic Fluency:** Say words starting with the letter "F" in one minute Score: Total number of animals and words listed (higher is better)  **Executive Function and Visual-spatial Organization (Trail Making Test B - TMT-B):** Draw line alternating between numbers (1-13) and letters (A-L) Score: Time taken to complete (lower is better, but multiplied by −1, so higher is better) |
| 29 | Gauci (2022), Australia | 1. Domain-specific cognitive functions (SUCCAB: Reaction and Decision Speed, Visual Processing, Spatial Working Memory, and Stroop Processing) | **Swinburne University Computerized Cognitive Assessment Battery (SUCCAB):**  **Simple Reaction Time:** Respond to a single white square with a right button press 30 targets with randomized intervals  **Choice Reaction Time:** Respond to a blue triangle or red square with left (blue) or right (red) button press 20 trials  **Immediate/Delayed Recognition:** Study 40 abstract images, then recognize them among 40 new images Indicate recognition with a right (yes) or left (no) button press Task repeated with new images at the end for delayed recognition  **Stroop Color–Word:** Congruent and incongruent Stroop task with color words Press corresponding button for ink color  **Spatial Working Memory:** 4x4 grid with six white squares Respond if subsequent squares match the original positions Total of 14 trials  **Contextual Memory:** 20 images of objects shown at various screen positions Recall original location with corresponding button press Assesses episodic memory  Performance on the eight tasks was grouped into four cognitive domains: Reaction and Decision Speed, Visual Processing, Spatial Working Memory, and Stroop Processing. |
| 30 | Huang (2022), China | 1. Global cognitive function (Chinese version MMSE) | The Chinese version of the Mini-Mental State Examination (MMSE) includes 24 questions spanning five dimensions: orientation, registration, attention and calculation, recall, and language. The MMSE total score is 30, with "cognitive impairment" defined as a score below 18 for illiterate individuals, below 21 for those with 1-6 years of education, and below 25 for those with more than 6 years of education. |
| 31 | Lawrie (2022), UK | 1. Global cognitive function (MoCA adjusted for education) | Cognitive impairment was evaluated using the Montreal Cognitive Assessment (MoCA), which was adjusted for educational levels. The adjusted total MoCA score was classified as a binary variable: scores below 26 indicated mild cognitive impairment, while scores of 26 or above indicated no mild cognitive impairment, based on MoCA guidelines. |
| 33 | Yeung (2022), China | 1. Domain-specific cognitive functions (Four items from MMSE: orientation to date, orientation to address, registration of three objects, attention and calculation) | Cognition was evaluated using the Mini-Mental State Examination (MMSE), consisting of 30 items. Four MMSE items with the highest communalities—'orientation to date,' 'orientation to address,' 'registration of three objects,' and 'attention and calculation'—were emphasized in the assessment. |
| 34 | Escher (2023), USA | 1. Domain-specific cognitive functions (Episodic memory; language functioning; executive functioning) | **Episodic Memory:** **California Verbal Learning Test (CVLT-II)** 16-item word list over five learning trials Free and cued recall after an interference trial Free recall after a 20-minute delay 44-word list to distinguish target words from distractors Defined by total words recalled after the long delay Sample-based z-scores estimated for better interpretation  **Language Functioning: Boston Naming Test (BNT-15)** Name 15 different objects **Category Fluency (animals)** Name as many animals as possible in 60 seconds Sample-based z-scores created and averaged for a composite score  **Executive Functioning: Stroop Interference Task** Name ink color of incongruent color words **Digit Span Backwards** Repeat digits in reverse order **DKEFS Design Fluency-Condition 1** Generate geometric patterns in 60 seconds **Modified Trail Making Test** Alternate between numbers and days of the week sequentially Sample-based z-scores estimated and averaged for a composite score |
| 35 | Zare (2023), Iran | 1. Global cognitive function (PCAP: FDST; LDMT; TMT; Stroop Task) | Persian paper and package of Pencil Cognitive Assessment Package (PCAP) Components:  **Working Memory:** Forward Digit Span Task (FDST)  **Executive Function:** Letter Digit Modality Task (LDMT) Letter/Symbol Digit Modalities Test  **Attention:** Trail Making Task (TMT) Stroop Task (ST) |
| 36 | Derdiger (2024), USA | 1. Global cognition function 2. Domain-specific cognitive functions  (CERAD; AFT; DSST) | **CERAD (Consortium to Establish a Registry for Alzheimer's Disease):** Assesses immediate learning and delayed recall. Presented with 10 words across three trials. Immediate Recall: Recall words immediately after hearing each set; individual trial scores averaged for final score. Delayed Recall: Recall words after completion of other tests (AFT and DSST).  **AFT (Animal Fluency Test):** Assesses categorical verbal fluency (executive function). Practice Test: Name three items of clothing; if unsuccessful, the test not administered. Final Score: Number of animals named in 1 minute.  **DSST (Digit Symbol Substitution Test):** Subtest within Wechsler Adult Intelligence Scale. Assesses processing speed, sustained attention, and working memory. Practice Test: Match symbols with numbers using a legend. Final Score: Total correct pairs identified within 2 minutes. Users unable to match without assistance do not proceed with test. |
| 37 | Arjmand (2022), Iran | 1. Domain-specific cognitive functions (FDST and BDST; LNST; SDMT; AVLT; TMT A&B; Stroop task) | **Verbal Short Memory Composite:** Forward Digit Span Task (FDST) Backward Digit Span Task (BDST)  **Working Memory Capacity:** Letter Number Sequencing Task (LNST)  **Attention and Visual Scanning:** Symbol Digit Modality Task (SDMT)  **Verbal Recognition Memory:** Auditory Verbal Learning Test (AVLT)  **Executive Function and Task Switching:** Trail Making Test A and B  **Cognitive Interference Inhibition:** Stroop Task |
| 38 | Barnes (2023), USA | 1. Global cognition function | Raw scores from each test were converted into z-scores using baseline mean and standard deviation values. These z-scores were then averaged across all tests to generate a global composite score. |
|  |  | 2. Domain-specific cognitive functions (Episodic memory; Semantic memory; Executive function; Perceptual speed) | **Episodic Memory:** Word List Memory Word List Recall Word List Recognition East Boston Story Immediate Recall East Boston Story Delayed Recall  **Semantic Memory:** Category Fluency (animals, fruits, and vegetables) Multilingual Naming Test  **Executive Function:** Trail Making Test B Flanker Inhibitory Control and Attention Test (NIH toolbox)  **Perceptual Speed:** Oral Symbol Digit Modality Test Pattern Comparison Test Trail Making Test A |
